# Ocean Currents Drove Genetic Structure of Seven Dominant Mangrove Species Along the Coastlines of Southern China

**DOI:** 10.3389/fgene.2021.615911

**Published:** 2021-03-08

**Authors:** Qifang Geng, Zhongsheng Wang, Jianmin Tao, Megumi K. Kimura, Hong Liu, Taizo Hogetsu, Chunlan Lian

**Affiliations:** ^1^School of Life Sciences, Nanjing University, Nanjing, China; ^2^Asian Natural Environmental Science Center, The University of Tokyo, Tokyo, Japan; ^3^College of Horticulture, Nanjing Agricultural University, Nanjing, China; ^4^Forest Tree Breeding Center, Forestry and Forest Products Research Institute, Ibaraki, Japan; ^5^Department of Earth and Environment, Florida International University, Miami, FL, United States; ^6^Graduate School of Agricultural and Life Sciences, The University of Tokyo, Tokyo, Japan

**Keywords:** conservation, gene flow, genetic diversity, mangrove, ocean current, population genetic structure

## Abstract

Mangrove forest ecosystems, which provide important ecological services for marine environments and human activities, are being destroyed worldwide at an alarming rate. The objective of our study was to use molecular data and analytical techniques to separate the effects of historical and contemporary processes on the distribution of mangroves and patterns of population genetic differentiation. Seven mangrove species (*Acanthus ilicifolius*, *Aegiceras corniculatum*, *Avicennia marina*, *Bruguiera gymnorrhiza*, *Kandelia obovata*, *Lumnitzera racemosa*, and *Rhizophora stylosa*), which are predominant along the coastlines of South China, were genotyped at nuclear (nSSR) and chloroplast (cpSSR) microsatellite markers. We estimated historical and contemporary gene flow, the genetic diversity and population structure of seven mangrove species in China. All of these seven species exhibited few haplotypes, low levels of genetic diversity (*H*_E_ = 0.160–0.361, with the exception of *K. obovata*) and high levels of inbreeding (*F*_IS_ = 0.104–0.637), which may be due to their marginal geographical distribution, human-driven and natural stressors on habitat loss and fragmentation. The distribution patterns of haplotypes and population genetic structures of seven mangrove species in China suggest historical connectivity between populations over a large geographic area. In contrast, significant genetic differentiation [*F*_ST_ = 0.165–0.629 (nSSR); *G*_ST_ = 0.173–0.923 (cpSSR)] indicates that populations of mangroves are isolated from one another with low levels of contemporary gene flow among populations. Our results suggest that populations of mangroves were historically more widely inter-connected and have recently been isolated, likely through a combination of ocean currents and human activities. In addition, genetic admixture in Beibu Gulf populations and populations surrounding Hainan Island and southern mainland China were attributed to asymmetric gene flow along prevailing oceanic currents in China in historical times. Even ocean currents promote genetic exchanges among mangrove populations, which are still unable to offset the effects of natural and anthropogenic fragmentation. The recent isolation and lack of gene flow among populations of mangroves may affect their long-term survival along the coastlines of South China. Our study enhances the understanding of oceanic currents contributing to population connectivity, and the effects of anthropogenic and natural habitat fragmentation on mangroves, thereby informing future conservation efforts and seascape genetics toward mangroves.

## Introduction

Mangrove forests form conspicuous wetland ecosystems, fringing extensive areas of coastlines in tropical and subtropical regions (Mitsch et al., 2009; Barbier et al., 2011). Approximately 80 species, from 20 plant families, have been recognized as characteristic of this plant community, primarily consisting of trees and shrubs that normally grow in the intertidal zone of marine coastal environments or estuarine margins (Duke, 1992). Mangrove forests are extremely productive ecosystems that provide important ecological services, both to the marine environment (e.g., coastal protection) and to industries including fisheries, timber and plant products; and tourism (Alongi, 2002; Barbier et al., 2011; Manez et al., 2014). Despite their ecological and economic importance, mangroves are being fragmented and destroyed worldwide at an alarming rate due to both climate change and human activity. They include sea-level fluctuations affected by climate change, urbanization, industrial pollution, farmland conversion, embankments for aquaculture ponds, and so on (Farnsworth and Ellison, 1997; Li and Lee, 1997; Ge and Sun, 2001; Stellman et al., 2003; Ong and Gong, 2013; Nguyen, 2014). This loss results in a serious ecological problem and requires urgent conservation in the face of further increases in climate fluctuation which is expected to have a dramatic effect on coastal areas (Ngeve et al., 2016; Yang et al., 2017). Recent phylogenetic and population genetics studies of *Rhizophora mucronata* in Southeast Asia, *Rhizophora racemosa* in East Atlantic, *Rhizophora mangle* in Florida, *Sonneratia alba* across the Indo-West-Pacific region, showed that historical events (e.g., climate change-induced sea level rise) and contemporary oceanographic conditions (e.g., surface currents) contributed to demographic histories and population genetic structure (Wee et al., 2014; Hodel et al., 2016; Ngeve et al., 2016; Yang et al., 2017). To better protect mangrove communities, a comprehensive understanding of the population dynamics and biographical processes associated with oceanic currents is necessary.

Ocean currents create a complex physical environment that influences dispersal, isolation, population connectivity and genetic structure of marine systems (Nakajima et al., 2014). Oceanic currents can act as gene-exchange corridors for the migration of marine organisms with long-distance dispersal (LDD) capability, and invisible physical barriers which may lead to genetic heterogeneity between adjacent populations (Palumbi, 1994; Gilg and Hilbish, 2003; Mitarai et al., 2009; Nakajima et al., 2014). Oceanic currents interact with historical events, environmental factors (e.g., temperature, salinity, and habitat availability) and biological traits (e.g., dispersal capability and life history) to determine species distribution range and phylogeographical structure in the sea (Nakajima et al., 2014; Li et al., 2017). Mangroves are characterized by a series of morphological and ecophysiological adaptations in thriving in tidally influenced areas (Van der Stocken and Menemenlis, 2017). Most mangroves have viviparous propagules which are buoyant and presumably capable of dispersal by ocean currents, which may play a role in the geographical distribution and population dynamics of mangrove plants (Yang et al., 2017). Mangroves are very dependent on LDD to allow for the colonization of unoccupied habitats, geographically distant areas and range expansion (Cain et al., 2000; Van der Stocken and Menemenlis, 2017). Therefore, it has been suggested that LDD and colonization can prevent speciation of geographically isolated populations (Cain et al., 2000). Several studies based on molecular markers have provided evidence that mangrove can detour around vast landmasses or across open seas and can effectively colonize geographically distant regions (Cain et al., 2000; Chiang et al., 2001; Liao et al., 2009; Takayama et al., 2013). In this study, we considered seven co-occurring mangrove species from five families [*Acanthus ilicifolius* (Acanthaceae), *Aegiceras corniculatum* (Myrsinaceae), *Avicennia marina* (Verbenaceae), *Bruguiera gymnorrhiza* (Rhizophoraceae), *Kandelia obovata* (Rhizophoraceae), *Lumnitzera racemosa* (Combretaceae), and *Rhizophora stylosa* (Rhizophoraceae)] across their entire distributional range in China. These seven species are dominantly distributed in the South China Sea (SCS). Owing to differences in pollination vectors and propagule types, these species are expected to have different potentials for gene flow. For this purpose, our main goals were to (i) investigate the patterns of genetic diversity and population genetic structure of seven dominant mangrove species with varying reproductive traits along the coastlines of southern China; (ii) examine whether and how oceanic currents along the South China Sea contributed to the biogeographic processes and population genetic structure of mangrove species. We then discuss the effects of major evolutionary processes, as well as the oceanic currents in the South China Sea that might have played dominant roles in shaping the current distribution and genetic patterns of mangrove populations. We further discuss the relative influence of historical versus contemporary processes on shaping the spatial distribution of genetic variation. This will allow us to assess the effects of anthropogenic habitat fragmentation on mangroves, thereby informing conservation efforts toward them.

## Materials and Methods

### Study Species

Seven species, which are common to the coastal mangroves of southeastern China, were included in this study (*A. ilicifolius*, *A. corniculatum*, *A. marina*, *B. gymnorrhiza*, *K. obovata*, *L. racemosa*, and *R. stylosa*). *Kandelia* spp. is mainly distributed along the coastlines of Asia and has a wide distribution from western and eastern India, through the South China Sea region to southern China and southern Japan (Tomlinson, 1986). *Kandelia* spp. has been divided into two species, one of which is still considered as *Kandelia candel* (distributed from the oceanic coast of Indian Ocean to southern South China Sea), and the other is named as *K. obovata* (distributed from northern South China Sea to southern Japan) (Sheue et al., 2003). Although these seven species are adapted to the environmental and ecological conditions of mangrove habitats, they still have different morphological attributes.

Three of the seven species (*B. gymnorrhiza*, *K. obovata*, and *R. stylosa*) are viviparous, two species (*A. corniculatum* and *A. marina*) are crypto-viviparous, and the remaining species (*A. ilicifolius* and *L. racemosa*) are non-viviparous (Ye et al., 2004a; Duke, 2006). The propagules of *A. corniculatum* and *L. racemosa* are not buoyant (Clarke, 1995), but the propagules of *A. corniculatum*, *B. gymnorrhiza*, *K. obovata*, and *R. stylosa* can survive in seawater for more than 3 months (Clarke et al., 2001; Ye et al., 2004b). For the other three species, the propagules can withstand seawater for shorter periods of time, varying between 11 days for *A. ilicifolius* and 3–4 weeks for *A. marina* (Steinke, 1986; Clarke et al., 2001).

### Sample Collection

**FIGURE 1:**
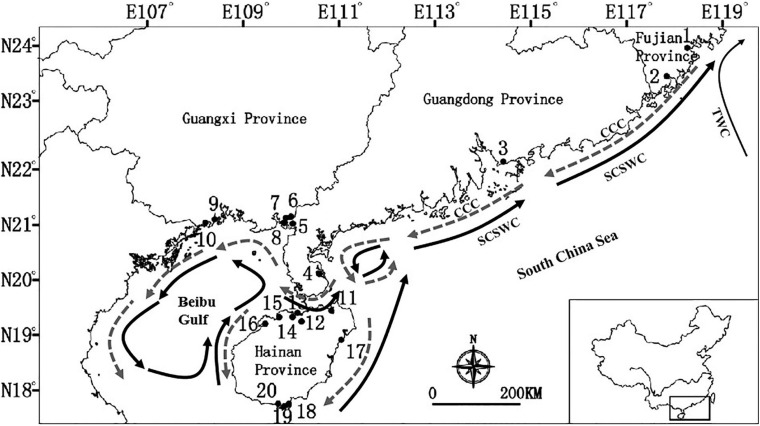
Sampling locations of the seven dominant mangrove species along the coastline of South China. Location identification numbers are given in brackets. See Table 1 for details of the number of species that were sampled in each of these 20 locations. Dark arrows are South China Sea Warm Current (SCSWC) and Taiwan Warm current (TWC) in the summer, and gray arrows is the China Coastal Current (CCC) in the winter in the South China Sea (Wang et al., 1991; Longhurst, 2007).

Our sampling targeted all mangrove communities throughout the coastlines of southeast China, including the provinces of Fujian, Hainan, Guangdong and Guangxi (Table 1). A total of 20 locations, as shown in Figure 1, were selected to collect samples. *R. stylosa* was present at 15 of these collection sites. In contrast, *A. corniculatum* and *L. racemosa* were only found at six of the locations. On average, 40 individuals of each species within each location were sampled, at an interval of at least 10 m. If the number of individuals at each location was less than 40, all of them were sampled. A total of 2,459 individuals were sampled for the seven mangrove species. The number of sampled individuals per population ranged between 8 and 49. All samples were immediately placed in plastic zip lock bags with silica gel for fast drying, and then stored at room temperature.

**TABLE 1:** Locations and sample number of the seven dominant mangrove species along the coastlines of South China (AI, *Acanthus ilicifolius*; AC, *Aegiceras corniculatum*; AM, *Avicennia marina*; BG, *Bruguiera gymnorrhiza*; KO, *Kandelia obovata*; LR, *Lumnitzera racemosa*; RS, *Rhizophora stylosa*).

Location	Coordinate	Number of samples						
		AI	AC	AM	BG	KO	LR	RS
**Fujian Province**								
(1) Jiulongjiang, Longhai City	24°26′57.0″N, 117°54′31.1″E	49	40	–	–	26	–	–
(2) Zhangjiang, Yunxiao City	23°55′31.2″N, 117°24′46.2″E	–	40	–	–	44	–	–
**Guangdong Province**								
(3) Futian, Shenzhen City	22°31′30.05″N, 114°00′03.52″E	20	20	–	20	20	–	20
(4) Jinji, Xuwen Prefecture	20°39′16.26″N, 110°22′29.72″E	–	40	40	–	37	–	25
(5) Deyao, Lianjiang Prefecture	21°31′59.14″N, 109°46′42.59″E	–	–	40	–	–	–	34
(6) Pohedi, Lianjiang Prefecture	21°34′02.20″N, 109°45′48.79″E	40	–	–	–	–	–	–
(7) Gaoqiao, Lianjiang Prefecture	21°33′57.02″N, 109°45′17.19″E	–	40	–	40	42	–	10
**Guangxi Province**								
(8) Shankou, Hepu City	21°33′31.29″N, 109°44′52.71″E	–	40	40	40	40	–	40
(9) Fangcheng, Fangchenggang City	21°38′38.33″N, 108°22′28″E	–	–	–	40	–	–	40
(10) Jiangping, Dongxing City	21°34′55.3″N, 108°09′56.3″E	–	9	40	40	28	–	–
**Hainan Province**								
(11) Dongzhai, Haikou City	19°57′02.8″N, 110°34′32.9″E	40	40	–	40	41	31	40
(12) Fushan, Chengmai Prefecture	19°54′22.8″N, 109°58′19″E	–	40	20	–	–	43	39
(13) Qiaotou, Chengmai Prefecture	19°57′20″N, 109°55′56″E	–	40	40	–	–	–	40
(14) Maniao, Lingao Prefecture	19°55′54.8N, 109°58′08.7″E	40	40	40	8	31	–	40
(15) Xinying, Lingao Prefecture	19°51′23.3″N, 109°34′05.7″E	–	40	40	–	–	22	40
(16) Danzhou, Danzhou City	19°43′32.2″N, 109°17′33.5″E	–	40	–	–	–	–	40
(17) Qinglan, Wenchang City	19°25′28.5″N, 110°45′08.1″E	40	40	–	40	22	40	40
(18) Tielu, Sanya City	18°15′44.1″N, 109°42′08.8″E	–	–	35	9	–	40	40
(19) Qingmei, Sanya City	18°13′53.1″N, 109°37′14.7″E	–	–	–	–	–	40	–
(20) Hekou, Sanya City	18°15′04.3″N, 109°30′51.3″E	–	–	34	–	–	–	40
Total populations		6	14	10	9	10	6	15
Total samples		229	509	369	277	331	216	528

### Nuclear and Chloroplast Microsatellite Genotyping

Total genomic DNA was extracted from dried leaves, using a modified cetyltrimethylammonium bromide method (Lian et al., 2003). The DNA was dissolved in 200 μL of sterilized water and stored at −30°C.

For microsatellite analysis, samples of *A. corniculatum*, *A. ilicifolius*, *A. marina*, *B. gymnorrhiza*, *K. obovata*, *L. racemosa*, and *R. stylosa* were genotyped with 10, 6, 11, 8, 10, 9, and 10 polymorphic nuclear microsatellite (nSSR) markers, respectively, and 7, 5, 13, 11, 8, 16, and 8 chloroplast microsatellite (cpSSR) markers, respectively. Details of these nuclear SSR (nSSR) and chloroplast SSR (cpSSR) markers are presented in Supplementary Table 1 (Maguire et al., 2000a; Sugaya et al., 2002; Islam et al., 2004, 2006a, b, 2008; Geng et al., 2006, 2007, 2008b, 2009; Geng, 2007). The PCR conditions for each nSSR and cpSSR locus are described in previous reports, as shown in Supplementary Table 1. The DNA fragment analyses followed the methods reported by Geng et al. (2009) and Lian et al. (2008).

### Genetic Diversity and Population Differentiation at Chloroplast Microsatellite Markers

To calculate chloroplast haplotype variation within populations for each species, the number of haplotypes (*n*_h_), number of polymorphic loci (*n*_p_), and Nei’s unbiased haplotype diversity estimates [*H*_E_; (Nei, 1987)] were computed using Arlequin version 3.5 (Excoffier and Lischer, 2010).

We calculated Nei’s unbiased genetic distance estimate for all population pairs [*G*_ST_; (Nei, 1978)], and Nei’s genetic differentiation index among populations, using the PopGene 1.32 program (Yeh and Boyle, 1997). The significance of correlations between genetic (*G*_ST_) and geographic distances, and between population pairs, was determined according to the Mantel test (Mantel, 1967), with 9,999 random permutations, using GenAlEx 6.5 software (Peakall and Smouse, 2006).

Population differentiation was estimated with Φ_ST_ and *N*_ST_ by using Permut & CpSSR version 2.0 (Pons and Petit, 1996; Burban et al., 1999) and significance tested with 10,000 permutations. Φ_ST_ is calculated only based on haplotype frequencies, however, *N*_ST_ also accounts for genetic distances between haplotypes. Phylogeographical structuring is usually indicated when *N*_ST_ value is higher than the Φ_ST_ values (10,000 permutations; *P* < 0.05) (Petit et al., 2005).

To determine the evolutionary relationships among haplotypes, a haplotype network based on the median-joining (MJ) algorithm was constructed using Network 5.0 (Bandelt et al., 1999).

### Genetic Diversity and Population Genetic Structure at Nuclear Microsatellite Markers

For genetic diversity levels of each species, according to nuclear SSR markers, the mean number of alleles per locus (*Ave*), expected (*H*_E_) and observed (*H*_O_) heterozygosity, and the number of private alleles (*P*_A_) for each population were estimated using GenAlEx 6.5 (Peakall and Smouse, 2006). The allelic richness (*R*) and inbreeding coefficients (*F*_IS_) were estimated using FSTAT version 2.9.4 (Goudet, 2003). The *deviation* of *F*_IS_ from zero was tested in each population by 1,000 permutation tests, with a sequential Bonferroni correction. Recent effective population sizes of each population (*N*_E_) were estimated using program ONeSAMP 2.0, which uses eight summary genetic statistics for approximate Bayesian computation (Tallmon et al., 2008). The lower and upper bounds of the prior distribution in all populations were two and 1,0000, respectively. Priors of 2–100 and 2–1,000 were also tested to assess whether the results were robust to changes in these assumed values. Analysis of molecular variance [AMOVA; (Excoffier et al., 1992)] was performed according to Arlequin version 3.5 (Excoffier and Lischer, 2010), with 1,000 permutations. We used all the populations of each species to estimate the variances attributable to differences between populations within species and among individuals within populations. The overall Wright’s *F*_ST_ values among all populations of each species were also estimated according to the Arlequin version 3.5 (Excoffier and Lischer, 2010). The pairwise *F*_ST_ (Weir and Cockerham, 1984) values between populations were estimated using GenAlEx6.5 (Peakall and Smouse, 2006). A random effects model, with 9,999 random permutations, was used to test the significance of each *F*_ST_ value.

To test isolation by distance, the relationship between genetic [*F*_ST_/(1 − *F*_ST_)] and geographical distances was assessed by Mantel test (Mantel, 1967), using GenAlEx 6.5 (Peakall and Smouse, 2006). Genetic distance was regressed against the straight-line geographic distances for all pairs of populations.

Bayesian methods were also used to recover the optimal number of clusters representing these populations. This analysis was performed using STRUCTURE 2.3 software (Pritchard et al., 2000). Twenty independent runs for each *K* (population numbers of each species) were performed using 1,000,000 Markov Chain Monte Carlo (MCMC) repetitions and 100,000 burn-in periods (Falush et al., 2003; Gilbert et al., 2012), no prior information, and assuming correlated allele frequencies and admixtures. The optimal *K* value was determined using the Δ*K* method of Evanno et al. (2005), as implemented in STRUCTURE HARVESTER (Earl and Vonholdt, 2012). To obtain the optimal alignment of the independent runs, the CLUMPP version 1.1 (Jakobsson and Rosenberg, 2007) was used, with 10,000 random input orders and 10,000 repeats, to calculate the average pairwise similarity (*H*′) of runs.

### Detection of Contemporary and Historical Gene Flow

We used BAYESASS 3.0.4 (Wilson and Rannala, 2003) and Migrate-N version 3.6 (Beerli and Palczewski, 2010), respectively, to estimate contemporary (past few generations) and historical gene flow (much longer period of time, approximately 4 *N*_e_ generations in the past (Beerli and Felsenstein, 2001; Beerli, 2006).

BAYESASS uses a Bayesian approach and a Markov chain Monte Carlo (MCMC) algorithm to estimate migration rates (*m*) between populations without assuming genetic equilibrium, which reflects gene flow over the most recent several generations (Wilson and Rannala, 2003). We ran 5 independent replicates with different random starting seeds for each species. We used 5 × 10^7^ iterations, with a burn-in of 5 × 10^6^ generations, and a sampling interval of 2000. We optimized the mixing parameters for migration rates (*m*), allele frequencies (*a*), and inbreeding coefficients (*f*) so that posterior acceptance rates for each parameter were between 20 and 60% as suggested by Wilson and Rannala (2003). For *A. corniculatum*, *A. ilicifolius*, *A. marina*, *L. racemosa*, and *R. stylosa*, we used *m* = 0.15, *a* = 0.4, and *f* = 0.5; for *B. gymnorrhiza* and *K. obovata*, we used *m* = 0.15, *a* = 0.4, and *f* = 0.7, respectively. Convergence of the MCMCs was checked by comparing the traces of each run using TRACER v. 1.7 (Rambaut et al., 2018). Among the five independent runs, we chose the one with the best consistency of the posterior distribution.

We used Migrate-N version 3.6 to investigate historical migration rates (*m*_h_) among populations (Beerli and Palczewski, 2010). MIGRATE uses a coalescent theory with MCMC to estimate effective population sizes (Theta = θ, θ = 4 *N*_e_μ, where *N*_e_ is historical effective population size and μ is mutation rate per generation) and the mutation-scaled migration rate (*M*) (*M* = *m*_h_/μ) between population pairs. Migrate-N assumes that populations are in migration-drift equilibrium, that population sizes and migration rates are constant through time, and that populations are randomly sampled. To estimate migration, we ran five replicates for each population in Migrate-N using Brownian motion mutation model with constant mutation rates and starting parameters based on *F*_ST_ calculations. The program estimates the parameters θ and *M* using a Bayesian method (Beerli and Felsenstein, 2001; Beerli, 2006). Both of which could be used to estimate the number of migrants per generation (*N*_m_) into each population using the equation 4 *N*_m_ = θ^∗^ *M*. We estimated θ and *M* with slice sampling and uniform prior distribution (for θ, range = 0–100.0, delta = 10; for *M*, range = 0–1000.0, delta = 100). Each of the five replicate runs was performed with 10,000-recorded genealogies at a sampling interval of 5 increments after discarding the first 100,000 genealogies (burn-in). Four-chain adaptive heating at temperatures (1.0, 1.5, 3.0, and 1,000,000) was used to increase the efficiency of the MCMC. The confidence interval for θ and the migration parameter *M* was calculated using a percentile approach (Beerli and Felsenstein, 2001). The mean value of *M* for each population pair in each direction was used.

## Results

### Genetic Diversity Within Populations

Based on the genetic diversity of the mangrove species, as estimated by cpSSR markers, *n*_h_ and *n*_p_ per locus per population ranged from 1.2 (*L. racemosa*) to 3.2 (*A. marina*), and from 0.3 (*A. ilicifolius*) to 2.1 (*K. obovata*), respectively (Table 2). Low levels of genetic diversity in cpSSRs were consistently observed in all species, as indicated by Nei’s unbiased haplotypic diversity (*H*_E_), ranging from 0.050 (*R. stylosa*) to 0.263 (*A. marina*; Table 2).

**TABLE 2:** Genetic diversity within different populations of the seven dominant mangrove species along the coastlines of South China as revealed by chloroplast microsatellite markers.

Population Species	Fujian		Guangdong					Guangxi			Hainan										
	1	2	3	4	5	6	7	8	9	10	11	12	13	14	15	16	17	18	19	20	Mean
***Acanthus ilicifolius***																					
*N*	49	–	20	–	–	40	–	–	–	–	40	–	–	40	–	–	40	–	–	–	38
*n*_h_	1.0	–	1.0	–	–	1.0	–	–	–	–	2.0	–	–	1.0	–	–	2.0	–	–	–	1.3
*n*_p_	0.0	–	0.0	–	–	0.0	–	–	–	–	1.0	–	–	0.0	–	–	1.0	–	–	–	0.3
*H*_e_	0.000	–	0.000	–	–	0.000	–	–	–	–	0.302	–	–	0.000	–	–	0.102	–	–	–	0.067
***Aegiceras corniculatum***																					
*N*	40	40	20	40	–	–	40	40	–	9	40	40	40	40	40	40	40	–	–	–	36
*n*_h_	1.0	1.0	1.0	1.0	–	–	1.0	3.0	–	3.0	1.0	1.0	2.0	1.0	1.0	2.0	1.0	–	–	–	1.4
*n*_p_	0.0	0.0	0.0	0.0	–	–	0.0	1.0	–	1.0	0.0	0.0	1.0	0.0	0.0	1.0	0.0	–	–	–	0.3
*H*_e_	0.000	0.000	0.000	0.000	–	–	0.000	0.099	–	0.556	0.000	0.000	0.054	0.000	0.000	0.051	0.000	–	–	–	0.054
***Avicennia marina***																					
*N*	–	–	–	40	40	–	–	40	–	40	–	20	40	40	40	–	–	35	–	34	37
*n*_h_	–	–	–	6.0	2.0	–	–	5.0	–	4.0	–	1.0	4.0	3.0	5.0	–	–	1.0	–	1.0	3.2
*n*_p_	–	–	–	4.0	1.0	–	–	2.0	–	2.0	–	0.0	1.0	1.0	3.0	–	–	0.0	–	0.0	1.4
*H*_e_	–	–	–	0.109	0.358	–	–	0.340	–	0.199	–	0.264	0.576	0.636	0.149	–	–	0.000	–	0.000	0.263
***Bruguiera gymnorhiza***																					
*N*	–	–	20	–	–	–	40	40	40	40	40	–	–	8	–	–	40	9	–	–	31
*n*_h_	–	–	1.0	–	–	–	1.0	2.0	2.0	2.0	2.0	–	–	1.0	–	–	2.0	2.0	–	–	1.7
*n*_p_	–	–	0.0	–	–	–	0.0	1.0	1.0	1.0	4.0	–	–	0.0	–	–	4.0	4.0	–	–	1.7
*H*_e_	–	–	0.000	–	–	–	0.000	0.050	0.050	0.050	0.142	–	–	0.000	–	–	0.235	0.556	–	–	0.120
***Kandelia obovata***																					
*N*	26	44	20	37	–	–	42	40	–	28	41	–	–	31	–	–	22	–	–	–	33
*n*_h_	1.0	5.0	3.0	4.0	–	–	4.0	2.0	–	5.0	2.0	–	–	1.0	–	–	3.0	–	–	–	3.0
*n*_p_	0.0	3.0	2.0	4.0	–	–	3.0	2.0	–	4.0	1.0	–	–	0.0	–	–	2.0	–	–	–	2.1
*H*_e_	0.000	0.145	0.389	0.119	–	–	0.264	0.512	–	0.263	0.049	–	–	0.000	–	–	0.176	–	–	–	0.192
***Lumnitzera racemosa***																					
*N*	–	–	–	–	–	–	–	–	–	–	31	43	–	–	22	–	40	40	40	–	36
*n*_h_	–	–	–	–	–	–	–	–	–	–	2.0	1.0	–	–	2.0	–	1.0	2.0	1.0	–	1.5
*n*_p_	–	–	–	–	–	–	–	–	–	–	4.0	0.0	–	–	2.0	–	0.0	1.0	0.0	–	1.2
*H*_e_	–	–	–	–	–	–	–	–	–	–	0.405	0.000	–	–	0.519	–	0.000	0.050	0.000	–	0.162
***Rhizophora stylosa***																					
*N*	–	–	20	25	34	–	10	40	40	–	40	39	40	40	40	40	40	40	–	40	35
*n*_h_	–	–	1.0	1.0	1.0	–	1.0	1.0	1.0	–	1.0	1.0	1.0	2.0	1.0	1.0	1.0	2.0	–	2.0	1.2
*n*_p_	–	–	0.0	0.0	0.0	–	0.0	0.0	0.0	–	0.0	0.0	0.0	6.0	0.0	0.0	0.0	2.0	–	2.0	0.7
*H*_e_	–	–	0.000	0.000	0.000	–	0.000	0.000	0.000	–	0.000	0.000	0.000	0.050	0.000	0.000	0.000	0.185	–	0.512	0.050

*N, number of individuals; n_*h*_, number of haplotypes; n_*p*_, number of polymorphic loci; H_*E*_, Nei’s unbiased haplotypic diversity.*

The mean number of alleles per locus (*Ave*) and allelic richness (*R*) per locus and per population, based on nSSR marker data, ranged from 1.8 (*R. stylosa*) to 7.2 (*K. obovata*), and 1.6 (*R. stylosa*) to 6.3 (*K. obovata*), respectively (Table 3). The observed (*H*_O_) and expected (*H*_E_) heterozygosities ranged from 0.088 (*R. stylosa*) to 0.544 (*K. obovata*), and 0.160 (*R. stylosa*) to 0.587 (*K. obovata*), respectively (Table 3). The lowest and highest genetic diversities were observed for *R. stylosa* and *K. obovata*, respectively. The mean values of recent effective population size (*N*_E_) ranged from 3.6 (*A. ilicifolius*) to 206.0 (*A. corniculatum*) (Table 3).

**TABLE 3:** Descriptive statistics over all nuclear microsatellite loci for each population of seven dominant mangrove species along the coastlines of South China.

Population Species	1	2	3	4	5	6	7	8	9	10	11	12	13	14	15	16	17	18	19	20	Mean
***Acanthus ilicifolius***																					
*N*	49	–	20	–	–	40	–	–	–	–	40	–	–	40	–	–	40	–	–	–	38
*Ave*	2.2	–	3.2	–	–	1.3	–	–	–	–	2.3	–	–	1.5	–	–	4.2	–	–	–	2.4
*R*	2.0	–	3.2	–	–	1.3	–	–	–	–	2.3	–	–	1.5	–	–	3.8	–	–	–	2.3
*H*_O_	0.130	–	0.267	–	–	0.046	–	–	–	–	0.788	–	–	0.025	–	–	0.550	–	–	–	0.301
*H*_E_	0.326	–	0.385	–	–	0.061	–	–	–	–	0.439	–	–	0.069	–	–	0.471	–	–	–	0.292
*F*_IS_	0.607*	–	0.330*	–	–	0.263	–	–	–	–	–0.791	–	–	0.645*	–	–	–0.156	–	–	–	0.150
*N*_E_	10.1	–	2.8	–	–	2.5	–	–	–	–	2.0	–	–	2.1	–	–	2.0	–	–	–	3.6
***Aegiceras corniculatum***																					
*N*	40	40	20	40	–	–	40	40	–	9	40	40	40	40	40	40	40	–	–	–	36
*Ave*	2.5	3.8	4.1	4.2	–	–	4.0	4.6	–	3.0	3.1	3.1	3.0	2.7	3.1	3.7	3.7	–	–	–	3.5
*R*	2.1	2.4	3.4	2.9	–	–	2.6	2.9	–	3.0	2.5	2.6	2.2	1.9	2.5	2.6	2.6	–	–	–	2.6
*H*_O_	0.259	0.208	0.365	0.373	–	–	0.320	0.363	–	0.533	0.378	0.268	0.258	0.200	0.385	0.293	0.305	–	–	–	0.322
*H*_E_	0.296	0.276	0.502	0.428	–	–	0.343	0.396	–	0.446	0.408	0.354	0.296	0.202	0.408	0.343	0.361	–	–	–	0.361
*F*_IS_	0.136*	0.259*	0.297*	0.143*	–	–	0.081*	0.096*	–	–0.139	0.087*	0.256*	0.142*	0.023	0.068	0.160*	0.167*	–	–	–	0.127
*N*_E_	12.9	492.3	18.9	183.4	–	–	160.6	369.3	–	19.8	52.5	134.3	15.2	36.2	21.9	124.6	71.2	–	–	–	122.4
***Avicennia marina***																					
*N*	–	–	–	40	40	–	–	40	–	40	–	20	40	40	40	–	–	35	–	34	37
*Ave*	–	–	–	3.1	2.7	–	–	2.8	–	3.4	–	2.5	2.8	3.5	3.2	–	–	2.4	–	2.5	2.9
*R*	–	–	–	2.9	2.6	–	–	2.6	–	3.2	–	2.5	2.6	3.1	2.9	–	–	2.3	–	2.3	2.7
*H*_O_	–	–	–	0.286	0.202	–	–	0.316	–	0.332	–	0.264	0.234	0.227	0.336	–	–	0.255	–	0.168	0.262
*H*_E_	–	–	–	0.380	0.295	–	–	0.364	–	0.442	–	0.379	0.273	0.334	0.370	–	–	0.330	–	0.310	0.348
*F*_IS_	–	–	–	0.259*	0.326*	–	–	0.143*	–	0.261*	–	0.328*	0.155*	0.331*	0.104*	–	–	0.243*	–	0.469*	0.262*
*N*_E_	–	–	–	45.8	76.3	–	–	44.2	–	472.1	–	6.6	70.6	483.7	137.9	–	–	7.0	–	7.4	135.2
***Bruguiera gymnorhiza***																					
*N*	–	–	20	–	–	–	40	40	40	40	40	–	–	8	–	–	40	9	–	–	31
*Ave*	–	–	2.4	–	–	–	2.5	2.4	2.6	2.9	2.6	–	–	2.3	–	–	3.5	2.9	–	–	2.7
*R*	–	–	1.9	–	–	–	2.1	2.2	2.2	2.1	1.6	–	–	2.0	–	–	2.5	2.7	–	–	2.1
*H*_O_	–	–	0.231	–	–	–	0.334	0.381	0.341	0.297	0.166	–	–	0.406	–	–	0.360	0.331	–	–	0.316
*H*_E_	–	–	0.301	–	–	–	0.358	0.378	0.374	0.352	0.167	–	–	0.350	–	–	0.426	0.499	–	–	0.356
*F*_IS_	–	–	0.257*	–	–	–	0.077	0.004	0.102*	0.169*	0.022	–	–	–0.096	–	–	0.166*	0.401*	–	–	0.122
*N*_E_	–	–	6.6	–	–	–	43.6	16.0	85.1	50.6	3.2	–	–	22.5	–	–	3.1	2.0	–	–	25.9
***Kandelia obovata***																					
*N*	26	44	20	37	–	–	42	40	–	28	41	–	–	31	–	–	22	–	–	–	33
*Ave*	6.6	8.2	8.0	7.9	–	–	8.7	8.8	–	8.6	5.6	–	–	6.0	–	–	3.2	–	–	–	7.2
*R*	6.2	6.7	8.0	6.7	–	–	7.4	7.4	–	7.7	4.7	–	–	5.3	–	–	3.1	–	–	–	6.3
*H*_O_	0.527	0.632	0.710	0.373	–	–	0.679	0.735	–	0.654	0.429	–	–	0.461	–	–	0.244	–	–	–	0.544
*H*_E_	0.596	0.670	0.696	0.548	–	–	0.711	0.689	–	0.667	0.459	–	–	0.485	–	–	0.347	–	–	–	0.587
*F*_IS_	0.135*	0.069*	0.006	0.331*	–	–	0.058*	–0.055	–	0.039	0.077*	–	–	0.065*	–	–	0.318*	–	–	–	0.104
*N*_E_	18.5	308.3	468.3	104.0	–	–	90.5	328.2	–	25.9	337.1	–	–	359.3	–	–	6.3	–	–	–	204.6
***Lumnitzera racemosa***																					
*N*	–	–	–	–	–	–	–	–	–	–	31	43	–	–	22	–	40	40	40	–	36
*Ave*	–	–	–	–	–	–	–	–	–	–	1.7	1.8	–	–	1.6	–	3.3	3.3	3.4	–	2.5
*R*	–	–	–	–	–	–	–	–	–	–	1.6	1.7	–	–	1.6	–	3.1	3.0	3.2	–	2.4
*H*_O_	–	–	–	–	–	–	–	–	–	–	0.057	0.063	–	–	0.035	–	0.142	0.150	0.167	–	0.102
*H*_E_	–	–	–	–	–	–	–	–	–	–	0.166	0.171	–	–	0.139	–	0.368	0.326	0.399	–	0.262
*F*_IS_	–	–	–	–	–	–	–	–	–	–	0.664*	0.640*	–	–	0.757*	–	0.623*	0.549*	0.591*	–	0.637*
*N*_E_	–	–	–	–	–	–	–	–	–	–	2.0	23.4	–	–	2.1	–	15.6	3.4	66.3	–	18.8
***Rhizophora stylosa***																					
*N*	–	–	20	25	34	–	10	40	40	–	40	39	40	40	40	40	40	40	–	40	35
*Ave*	–	–	1.3	1.9	1.7	–	1.3	2.1	2.2	–	1.6	1.6	2.1	2.3	1.7	1.9	1.6	2.3	–	2.1	1.8
*R*	–	–	1.2	1.9	1.6	–	1.3	1.6	1.7	–	1.4	1.4	1.9	1.7	1.6	1.7	1.5	2.0	–	1.7	1.6
*H*_O_	–	–	0.035	0.144	0.047	–	0.060	0.070	0.078	–	0.043	0.028	0.073	0.081	0.123	0.078	0.078	0.223	–	0.156	0.088
*H*_E_	–	–	0.062	0.257	0.141	–	0.077	0.127	0.155	–	0.142	0.077	0.223	0.175	0.171	0.170	0.171	0.268	–	0.182	0.160
*F*_IS_	–	–	0.455*	0.457*	0.675*	–	0.270	0.457*	0.509*	–	0.706*	0.643*	0.682*	0.549*	0.294*	0.552*	0.556*	0.183*	–	0.157*	0.476*
*N*_E_	–	–	3.9	2.5	32.2	–	2.1	134.2	2714.9	–	2.1	2.3	2.0	9.6	14.0	110.4	4.0	9.9	–	46.0	206.0

N, number of individuals; Ave, number of alleles per locus; R, allelic richness; H_*O*_, observed heterozygosity; H_*E*_, expected heterozygosity; F_*IS*_, inbreeding coefficient; *, significant deviation from zero (P < 0.05); N_*E*_, effective population size.

Allelic richness (*R*) and expected heterozygosity (*H*_E_) data are shown in Supplementary Figure 1 and they suggest that levels of genetic diversity of the seven species were independent of location. Therefore, species co-occurring in a particular site displayed different *R*, *H*_E_, and *P*_A_ values (Supplementary Figure 1). Most of the inbreeding coefficients were positive, ranging from 0.104 (*K. obovata*) to 0.637 (*L. racemosa*), indicating that most of these mangrove populations experienced at least moderate levels of inbreeding (Table 3).

### Genetic Differentiation Between Populations and Population Structure Estimation

The analysis of genetic differentiation between populations, based on cpSSR data, showed a strong population structure in each mangrove species throughout the coastlines of southeast China. The highest estimate of genetic differentiation (*G*_ST_) among the populations was for *R. stylosa* (0.923), followed by *A. ilicifolius* (0.834), *L. racemosa* (0.787), *A. marina* (0.530), *K. obovata* (0.438), *B. gymnorrhiza* (0.274), and *A. corniculatum* (0.173). Significant correlations, between geographical distance and genetic distance (*G*_ST_) estimates, were observed for *A. marina* and *K. obovata* (*P* < 0.01), but not among the other five species (*P* = 0.109 to 0.443; Supplementary Figure 2).

Hierarchical AMOVA of nSSR data for the seven species showed that most of the genetic variation was among populations within *A. ilicifolius* (62.9%), *L. racemosa* (50.7%), and *R. stylosa* (58.0%), while, for the other four species, most of the genetic variation was detected within populations (*A. corniculatum*, 72.9%; *A. marina*, 75.1%; *B. gymnorrhiza*, 83.5%; and *K. candle*, 79.9%; Table 4). The *F*_ST_ values computed across populations for each species, based on nSSR data, ranged from 0.165 (*B. gymnorrhiza*) to 0.629 (*A. ilicifolius*), which indicated a high level of genetic differentiation among populations for these species (Table 4). The pairwise *F*_ST_ values, between populations and within the seven species, ranged from 0.251 to 0.822 (*A. ilicifolius*), 0.025 to 0.373 (*A. corniculatum*), 0.040 to 0.314 (*A. marina*), 0.004 to 0.254 (*B. gymnorrhiza*), 0.015 to 0.351 (*K. candle*), 0.165 to 0.579 (*L. racemosa*), and 0.007 to 0.747 (*R. stylosa*; Supplementary Table 2), which further confirmed that genetic differentiation among the populations was extremely high within all of the species. No significant relationships, between geographical and genetic distance, were observed from the nSSR analysis (*P* = 0.089–0.607), with the exception of *A. corniculatum* (*P* = 0.0001; *R*^2^ = 0.622; Supplementary Figure 3).

**TABLE 4:** Hierarchical analysis of molecular variance (AMOVA), overall Wrights’s *F*_ST_ and number of migrants per generation (*N*_m_) of the seven dominant mangrove species along the coastlines of South China estimated by nuclear microsatellite markers.

Source of variation	d.f.	SS	Variance components	Percentage of variation	*F*_ST_	*N*_m_	*P*-value
***Acanthus ilicifolius***							
Among populations	5	557.406	1.465	62.9	0.629	0.147	<0.001
Within populations	452	390.426	0.864	37.1			<0.001
Total	457	947.832	2.328				
***Aegiceras corniculatum***							
Among populations	13	643.816	0.660	27.1	0.270	0.676	<0.001
Within populations	1004	1781.049	1.774	72.9			<0.001
Total	1017	2424.864	2.434				
***Avicennia marina***							
Among populations	9	442.023	0.641	24.9	0.245	0.770	<0.001
Within populations	728	1408.313	1.935	75.1			<0.001
Total	737	1850.336	2.576				
***Bruguiera gymnorrhiza***							
Among populations	8	143.468	0.275	16.5	0.163	1.284	<0.001
Within populations	545	758.183	1.391	83.5			<0.001
Total	553	901.652	1.666				
***Kandelia obovata***							
Among populations	9	476.554	0.760	20.1	0.199	1.006	<0.001
Within populations	652	1966.101	3.015	79.9			<0.001
Total	661	2442.656	3.775				
***Lumnitzera racemosa***							
Among populations	5	464.661	1.284	50.7	0.497	0.253	<0.001
Within populations	426	531.823	1.248	49.3			<0.001
Total	431	996.484	2.532				
***Rhizophora stylosa***							
Among populations	14	1149.740	1.160	58.0	0.573	0.186	<0.001
Within populations	1041	874.099	0.840	42.0			<0.001
Total	1055	2023.839	2.000				

**FIGURE 2:**
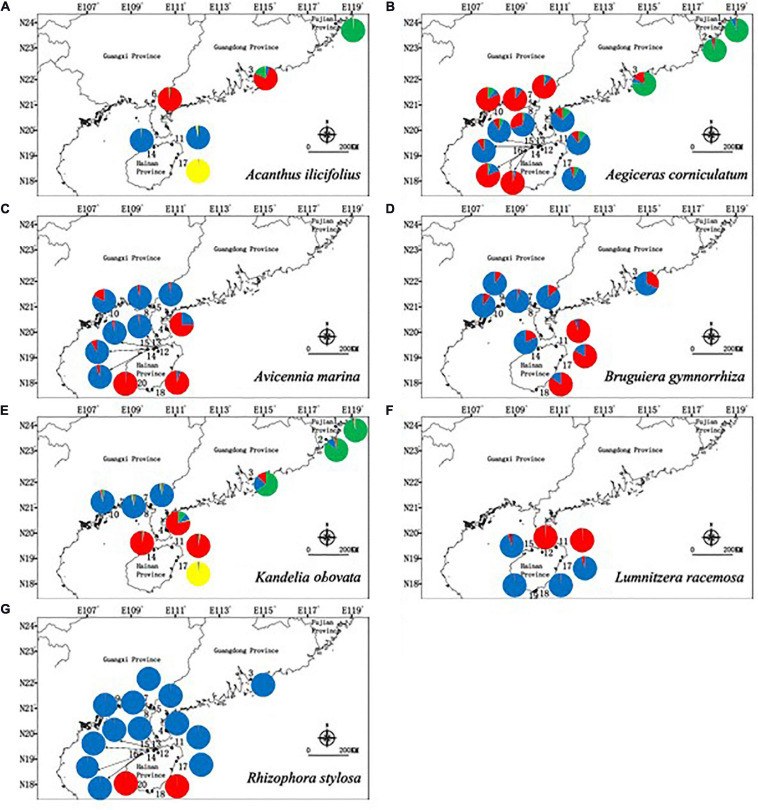
Geographic distribution of genetic groups detected from a STRUCTURE analysis of **(A)** *Acanthus ilicifolius* (Δ*K* = 4), **(B)** *Aegiceras corniculatum* (Δ*K* = 3), **(C)** *Avicennia marina* (Δ*K* = 2), **(D)** *Bruguiera gymnorrhiza* (Δ*K* = 2), **(E)** *Kandelia obovata* (Δ*K* = 4), **(F)** *Lumnitzera racemosa* (Δ*K* = 2), and **(G)** *Rhizophora stylosa* (Δ*K* = 2).

The inferred best *K* values from STRUCTURE cluster analysis were different between the species, with *K* = 4 clusters for *A. ilicifolius*, 3 clusters for *A. corniculatum*, 2 clusters for *A. marina*, 2 clusters for *B. gymnorrhiza*, 4 clusters for *K. obovata*, 2 clusters for *L. racemosa*, and 2 clusters for *R. stylosa*, respectively (Figure 2 and Supplementary Figure 4). These results showed that although different species aggregated in different clusters, populations within a similar geographical distance had similar genetic structures.

### Distribution of Haploytpes and Phylogeography

**FIGURE 3:**
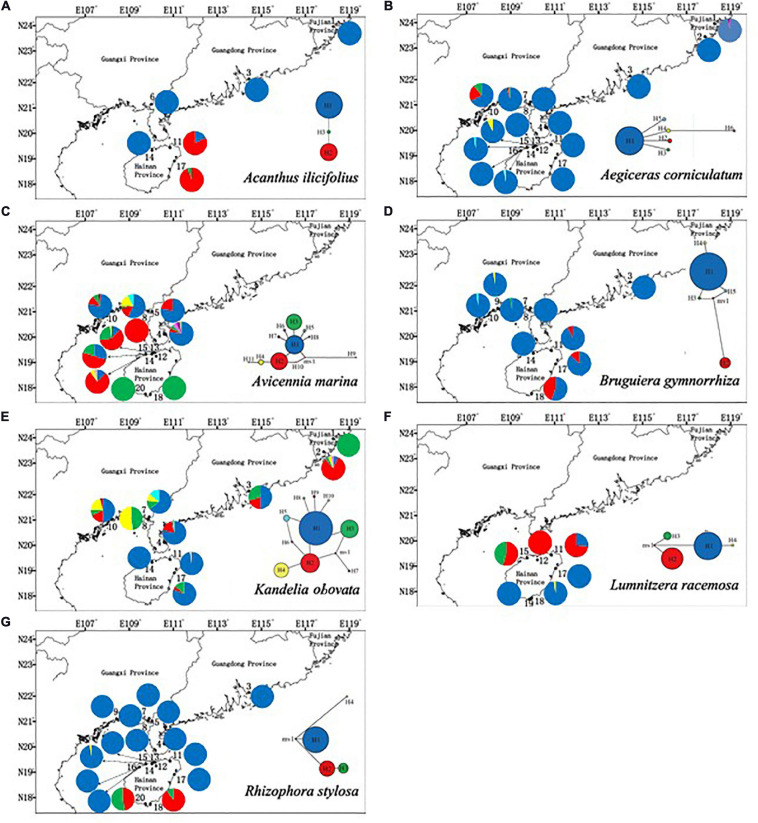
Geographic distribution of cpSSR haplotypes of **(A)** *Acanthus ilicifolius* (three haplotypes), **(B)** *Aegiceras corniculatum* (five haplotypes), **(C)** *Avicennia marina* (11 haplotypes), **(D)** *Bruguiera gymnorrhiza* (five haplotypes), **(E)** *Kandelia obovata* (10 haplotypes), **(F)** *Lumnitzera racemosa* (four haplotypes), and **(G)** *Rhizophora stylosa* (four haplotypes).

The polymorphic cpSSR markers yielded 3 to 11 haplotypes among the seven mangrove species. The highest number of haplotypes was found in *A. marina* (11). For all of the species, only a few haplotypes were detected in most of their populations (one type in *A. ilicifolius*, *A. corniculatum*, *B. gymnorrhiza*, *K. obovata*, and *R. stylosa*; two types in *L. racemosa*; and three types in *A. marina*). The remaining haplotypes were found in a few locations. Among seven mangrove species, significant phylogeographic structure was only observed in *R. stylosa* [*N*_ST_ (0.921) > Φ_ST_ (0.805), *P* < 0.05] (Figure 3 and Supplementary Table 3).

### Contemporary and Historical Gene Flow

The contemporary migration rates estimated in BAYESASS were low (*m* < 0.1) for most population pairs of all seven mangrove species (Supplementary Table 4). However, the number of migrants per generation (*N*_m_) for historical times as calculated by Migrate-N were high (*N*_m_ > 1) in seven mangrove species (Supplementary Table 5). Estimates of contemporary gene flow of seven mangrove species were lower than the historical times, which suggest that gene flow might be affected by recent habitat loss and fragmentation of these mangrove species.

**FIGURE 4:**
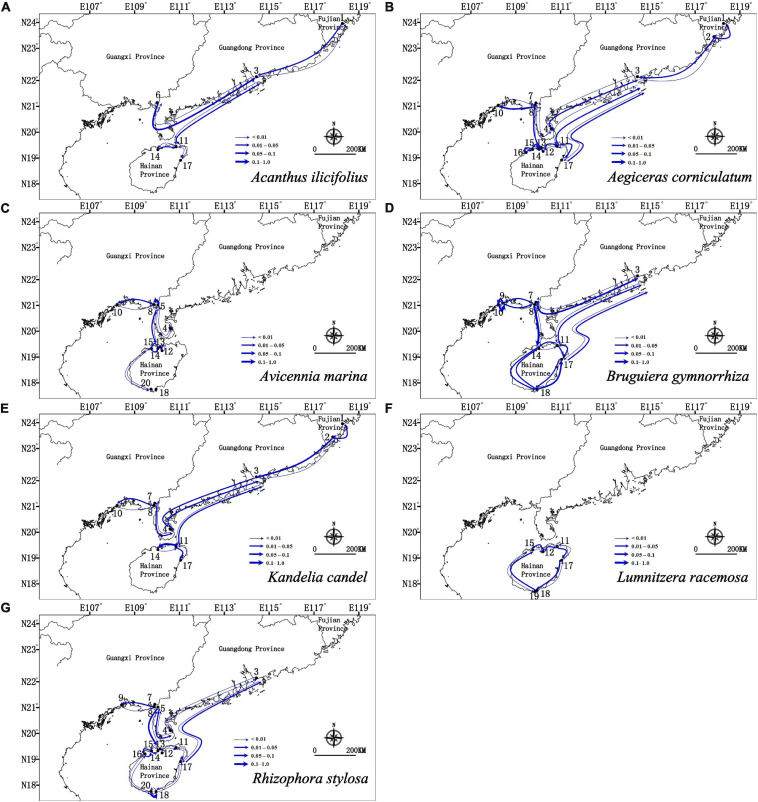
Contemporary gene flow estimated by BAYESASS between populations of seven dominant mangrove species along the coastline of South China based on nuclear SSR analysis. The width of each arrow is proportional to the relative level of gene flow.

**FIGURE 5:**
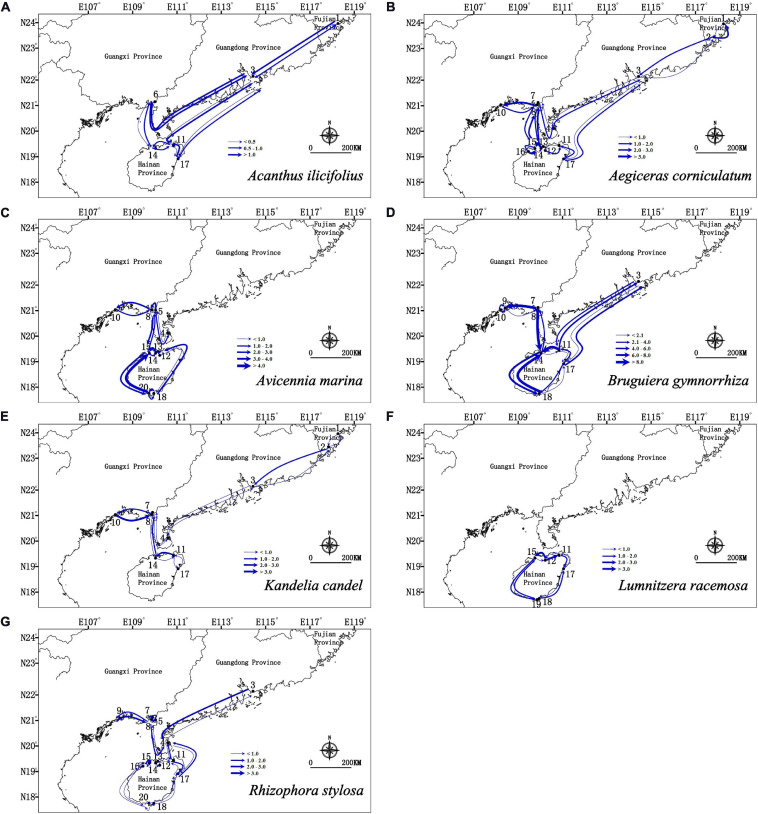
Gene flow estimated by Migrate-N between populations of seven dominant mangrove species based on nuclear SSR analysis. The direction of gene flow shown by the levels of *N*_m_ (the effective number of migrants per generations). The width of each arrow is proportional to the relative level of gene flow.

Migration analyses inferred from BAYESASS and Migrate-N congruently revealed the similar asymmetric gene flow in all seven mangrove species along the coastlines of South China (Supplementary Tables 4, 5 and Figures 4, 5), and which also revealed that contemporary gene flow was much lower than the historical gene flow. For populations surrounding the Hainan Island and mainland China, strong directional differences in gene flow was detected from populations in eastern Hainan Island (11–17) to the eastern coastlines of South China (3, 4) in four species (*A. ilicifolius*, *A. corniculatum*, *B. gymnorrhiza*, and *R. stylosa*; Figures 1, 4, 5 and Supplementary Tables 4, 5). For all seven mangrove species, highly asymmetric gene flows were detected from south to north, almost two to thirty-seven times larger than that in the opposite direction, which was congruent with the direction of seasonal marine currents (Figures 1, 4, 5 and Supplementary Tables 4, 5). For northern populations near the Taiwan Strait, strong asymmetric gene flow was detected in five species (*A. ilicifolius*, *A. corniculatum*, *B. gymnorrhiza*, *K. obovata*, and *R. stylosa*) (Supplementary Tables 4, 5 and Figures 4, 5). For populations along Beibu Gulf (5–8) and Qiongzhou Strait (11–16), symmetric or asymmetric gene flow was bidirectional (Supplementary Tables 4, 5 and Figures 4, 5). Furthermore, strong directional differences in gene flow were detected between adjacent populations in Beibu Gulf, i.e., gene flow from population 8–10 was ten times higher than the opposite direction in *A. corniculatum*, *A. marina*, and *K. obovata* (Supplementary Tables 5, 6 and Figures 4, 5). Strong directional differences in gene flow were detected in seven species, respectively (highlighted in the Supplementary Tables 4, 5 and Figures 4, 5). In addition, recent and historical gene flow, as assessed in BAYESASS and Migrate-N, was all lowest in *A. ilicifolius*, and highest in *B. gymnorrhiza* of the seven mangrove species (Supplementary Tables 4, 5).

## Discussion

### Genetic Diversity of Seven Mangrove Species

Accurate estimates of genetic diversity are very useful for optimizing sampling strategies, and for conserving and managing the genetic resources of trees (Hamrick et al., 1991; Schaal et al., 1991; Chalmers et al., 1992; Cardoso et al., 1998). The current distribution of mangrove species in China is located in the most northerly boundary of mangroves distribution worldwide. Nuclear SSR markers revealed low levels of genetic diversity in the populations of most mangrove species in China (*H*_E_ from 0.160–0.361), with the exception of *K. obovata* (*H*_E_ = 0.587). High levels of inbreeding (*F*_IS_ = 0.104–0.637) were also detected in most populations. Small recent effective population sizes for all mangrove species (3.6–206.0) were also detected. The cpSSR markers identified only a few haplotypes that were found in most populations, while the remaining haplotypes only occurred in a few populations (Figure 3).

Low genetic diversity might arise from population substructures or founder events, in which locally, high level of inbreeding have led to demes of genetically similar individuals (Tan et al., 2005). During interglacial periods, the founder effect during recolonization led to a further decrease in the genetic diversity of peripheral populations, such as *A. marina* and *Hibiscus tiliaceus* (Takayama et al., 2006). The low level of genetic diversity of most mangrove species in our study, was also reported in some previous molecular studies of mangroves along the coastlines of China, such as *B. gymnorrhiza* (Ge et al., 2005) and *Ceriops tagal* (Ge and Sun, 2001). These results may support the central-marginal hypothesis (Sagarin et al., 2006), which predicts lower genetic diversity and higher genetic differentiation in marginal populations (Eckert et al., 2008). A similar trend in the genetic characteristics between marginal and central populations was revealed in widely distributed mangrove species, such as *A. marina* (Maguire et al., 2000b; Arnaud-Haond et al., 2006), *Excoecaria agallocha* (Zhang et al., 2008), *R. mangle* (Sandoval-Castro et al., 2012), and *S. alba* (Yang et al., 2017). Peripheral populations may undergo consequences of bottlenecks, founder effects, reduced gene flow, strong genetic drift, low effective population size, inbreeding and high environmental stress, which would lead to lower genetic diversity and higher genetic differentiation than in central populations (Eckstein et al., 2006).

Furthermore, nearly two-thirds of the mangrove community in China were destroyed by industrialization, urbanization and coastal development during the past century (Li and Lee, 1997). As a result, the habitat area of mangroves is drastically decreased and discontinuous. Fragmentation effects on species more strongly at the periphery of their distributions compared to the core, which will lead to the loss of genetic variation, the increase of genetic differentiation between populations and the decrease of gene flow (Henle et al., 2017).

Lakshmi et al. (1997) suggested that different mangrove species are likely to display varying degrees of polymorphism, depending on their edaphic preferences and adaptations. We identified species-specific patterns of genetic diversity for the mangrove species included in our study (Supplementary Figure 1), indicating that the genetic diversity of every species might not be location-dependent, but was rather attributed to species-specific differences in their history of establishment and physiological traits.

### Population Genetic Structure of Seven Mangrove Species

The South China Sea (SCS) is one of the world’s most diverse marine ecosystems (Trajano et al., 2017), and is a region comprised of diversified mangrove species. The current distribution of mangrove species in China is confined to the northern coastlines of the SCS, and it represents the most northern boundary of worldwide distribution of mangroves. Along the coastlines of southern China, there is the northward flowing South China Sea Warm Current (SCSWC) which converges with the Taiwan Warm current (TWC) in the East China Sea in the summer (Figure 1). As mangrove global discontinuous distributions (Duke et al., 2002), and it has been assumed that buoyant propagules can be passively dispersed by ocean currents over long distances, because of their buoyancy, longevity, and water-dispersal (Clarke, 1993; Steinke and Ward, 2003; Nathan, 2006). However, a long-distance dispersal event is rare, stochastic and complex (Nathan, 2006), which acts as a bridge to connect isolated and distant populations, and potentially contributing to a chaotic genetic pattern for mangroves at a regional level. Here, our integrative evidence suggests that the population genetic structures of seven dominant mangrove species were mainly shaped by oceanic currents along the coastlines of southern China, especially in historical times.

The patterns of cpSSR haplotype distribution and population genetic structures of the seven mangrove species showed that although different species aggregated in different clusters, populations within a similar geographical distance had similar genetic structures (Figures 2, 3), which suggests that propagule dispersal of the mangrove species mostly occurred within a mesoscale range. The importance of considering ocean current patterns in explaining the genetic structure of coastal ecosystems has also been postulated by Wee et al. (2014), who demonstrated that genetic discontinuity between mangrove populations of *R. mucronata* is maintained by ocean features. Stocken et al. (2019b) also found high rates of along-coast transport and transoceanic dispersal in mangroves across the Atlantic, Pacific and Indian oceans. The trend of historical gene flow follows the direction of ocean currents in mangroves in northern SCS (Supplementary Table 5 and Figure 5), which suggests that the primary ocean currents can drive population genetic connectivity of these seven mangroves in this region in historical times.

First, mangrove populations in Beibu Gulf were mainly affected by the ocean currents (Figures 1, 5). The Beibu Gulf (Gulf of Tonkin), is located in the northwest South China Sea. At the surface, the residual flow direction during winter is basically westward or southwestward, while during summer it is almost reversed in Beibu Gulf (Manh and Yanagi, 2000). A certain degree of genetic connectivity was evident among the populations of Beibu Gulf (5–8) and Qiongzhou Strait (11–16) for all mangrove species, based on both haplotype distributions and Bayesian clustering analysis (Figures 2, 3), which is not consistent with our expectation. Oceanic current-mediated strong asymmetric historical gene flow (*N*_m_ > 1) between the populations in the northeast Beibu Gulf around Hainan Island (11–16) and the northern Beibu Gulf (5–8) for five mangrove species were found (Supplementary Table 5 and Figure 5). The oceanic current might lead to a high level of population genetic homogeneity of mangroves across a mesoscale geographical range in historical times. Our results showed that historical gene flow affected the current genetic structure of mangroves to some extent. In addition, strong directional differences in gene flow were detected between adjacent populations in northern Beibu Gulf, i.e., gene flow from population 8 to 10 was ten times higher than the opposite direction in *A. corniculatum*, *A. marina*, and *K. obovata* (Supplementary Table 5 and Figure 5), suggesting that oceanic currents in Beibu Gulf contributed strongly to population connectivity of mangroves in this gulf.

Second, populations of mangroves surrounding the Hainan Island and mainland China were mainly affected by the SCSWC (Figure 1) (Wang et al., 1991). A strong directional differences in gene flow was detected from populations in eastern Hainan Island (11, 17) to the eastern coastlines of South China (3, 4) in four species (*A. ilicifolius*, *A. corniculatum*, *B. gymnorrhiza*, and *R. stylosa*), over 450 km across the Qiongzhou strait (Figures 1, 5 and Supplementary Table 5). The detected gene flow along the path of SCSWC was almost 2–37 times larger than that in the opposite direction (Supplementary Table 5 and Figure 5), suggesting that the SCSWC contributed strongly to population genetic structure of mangroves in the northern SCS. In addition, near the Taiwan Strait highly asymmetric gene flow was detected from south to north (from population 2 to population 1) along the SCSWC and TWC in *A. corniculatum* and *K. obovata* (Figure 5). Such a dispersal pattern has also been observed in seagrass, *Sargassum thunbergii* (Li et al., 2017), *Sargassum horneri* (Hu et al., 2011), and *Sargassum fusiforme* (Hu et al., 2013) in northern SCS, which suggest that the northward running SCSWC and TWC have had more profound impacts on phylogeographical structure of marine species along the China coasts than the southward flowing China Coastal Current (Li et al., 2017).

Our results contribute to the growing evidence that ocean circulation plays important roles in population genetic connectivity in mangroves in the coastlines of southern China. Similar phenomenon was also found in *Rhizophora apiculata* in the Indo-Malesian region (Guo et al., 2016), *R. mangle* populations in Brazil and Florida (Pil et al., 2011; Hodel et al., 2016), *R. mucronata* populations in Southeast Asia (Wee et al., 2014), and *R. racemosa* in Eastern Atlantic (Ngeve et al., 2016).

Furthermore, the extent of gene flow varied among each mangrove species. The quantification of the numbers of propagules available for dispersal, the timing of propagule release in combination with the tidal framework and seasonal changes in ocean circulation, propagule buoyancy, viability period of propagules in seawater are the potential factors for the mangrove propagule dispersal (Stocken et al., 2019a). Among these factors, dispersal distance and connectivity of mangroves may be highly sensitive to the minimum and maximum floating periods (Stocken et al., 2019b). Recent and historical gene flow, as inferred from BayesAss and Migrate-N, was congruent lowest in *A. ilicifolius*, while highest in *B. gymnorrhiza* among seven mangrove species (Supplementary Tables 4, 5). The gene flow comparisons from BayesAss and Migrate-N may mean that the larger, longer-lived propagules of *B. gymnorrhiza* are more successful in dispersing long distance via ocean currents than the smaller, shorter-lived and no buoyancy propagules of *A. ilicifolius* (Steinke, 1986; Clarke et al., 2001; Ye et al., 2004b; Stocken et al., 2019b), which was further supported by genetic differentiation in these two species. The *F*_ST_ values of *A. ilicifolius* (0.629) were higher than for *B. gymnorrhiza* (0.165).

### Impact of Habitat Fragmentation on Seven Mangrove Species

Mangroves are being fragmented and destroyed, which were attributed primarily to human activities (Ngeve et al., 2016; Goldberg et al., 2020). As a result, mangroves were expected to experience erosion of genetic variation, reduced population size and increased population genetic differentiation and reduced gene flow.

In the present study, the contemporary effective population size (*N*_E_) ranged from 3.6 (*A. ilicifolius*) to 206.0 (*A. corniculatum*) (Table 3). This is consistent with increasing human activities, which led to a dramatic reduction in the number of habitats and the effective population size of mangroves (Barber et al., 2014; Yang et al., 2017). During a reduction in population size, the range in allele size would be expected to decrease as quickly as the number of alleles (Garza and Williamson, 2001), and as a result genetic diversity is lost very rapidly, which could explain the low genetic diversity of most mangroves in present study.

In the present study, both the nSSR and cpSSR markers revealed the relative high genetic differentiation among populations of the seven species [overall *F*_ST_ = 0.165-0.629 (nSSR); *G*_ST_ = 0.173-0.923 (cpSSR)] according to the AMOVA, especially the *A. ilicifolius*, *L. racemosa* and *R. stylosa* populations (Table 4). Similar results were obtained for previous mangroves studies in China, such as *Heritiera littoralis* (*G*_ST_ = 0.238) (Jian et al., 2004), *C. tagal* (*G*_ST_ = 0.529) (Ge and Sun, 2001), *K. obovata* (*G*_ST_ = 0.5548) (Chen et al., 2010). Furthermore, most of the pairwise *F*_ST_ values between populations within seven mangroves were also significantly high (*P* < 0.05, Supplementary Table 2). The high level of genetic differentiation seems to be the result of the reduced population size that induced the genetic drift.

In keeping with the above, relative low contemporary gene flow among most population pairs of all seven mangrove species (*m* < 0.1) was lower than historical gene flow (*N*_m_ > 1) (Supplementary Tables 4, 5). This finding shows that the recent habitat fragmentation in these mangroves populations is accompanied by decreased gene flow. This conclusion was confirmed by several studies in mangroves, such as *A. corniculatum* (Ge and Sun, 1999; Deng et al., 2009), *C. tagal* (Ge and Sun, 2001; Liao et al., 2011), *H. littoralis* (Jian et al., 2004; Jian S.-G. et al., 2010), *K. candel* (Huang, 1994; Sun et al., 1998; Chiang et al., 2001; Geng et al., 2008a), *L. racemosa* (Su et al., 2006), and *Nypa fruticans* (Jian S. et al., 2010). Reduced contemporary gene flow can further decrease genetic diversity and contribute to high levels of genetic differentiation in these mangrove species, which is consistent with the conclusions above. Although, the trend of recent gene flow follows the direction of ocean currents in mangroves in northern SCS, it is too little to offset the dramatic habitat fragmentation in the short term.

## Conclusion

Overall, we found that most mangrove species in China have low levels of genetic variation within populations, except for *K. obovata*. Local population extinction could result in a decreased potential to adapt to environmental changes, through the loss of alleles with adaptive value. In addition, a reduction in the number of populations and their sizes could enhance genetic drift and inbreeding, leading to reduced fitness. The conservation of mangrove genetic resources involves not only preventing extinction, but also ensuring the preservation of alleles that could provide adaptive values in changing environments (Namkoong, 1997; Tan et al., 2005).

The patterns of cpSSR haplotype distribution, population genetic structure and strong asymmetric historical gene flow of the seven mangrove species along the coastlines of China, suggests that populations of mangroves were mainly driven by oceanic currents in historical times. In contrast, high genetic differentiation indicates that populations of mangroves are isolated with low levels of contemporary gene flow, which might be due to natural and anthropogenic habitat fragmentation. The recent isolation and lack of gene flow among populations of mangroves may affect their long-term survival along the coastlines of South China. Our results showed that the strong and marked genetic differentiation among populations of each of the species resulted in several geographically distinct genetic clusters, indicating the existence of genetically-similar bioregions. Four bioregions (Fujian, Guangdong, Guangxi, and Hainan) could be identified based on genetic structure results, and any activities resulting in gene mixing with each other ought to be prevented. We suggest that future ecological restoration initiatives should establish these bioregions as conservation units. Within the different bioregions of each species, plants could be transplanted to diminish the risk of fitness decline and loss of genetic diversity of the native plant populations.

## Data Availability Statement

The raw data supporting the conclusions of this article will be made available by the authors, without undue reservation.

## Author Contributions

QG, CL, and TH conceived the study. QG performed the experiments, analyzed the data, and wrote the manuscript. JT helped to collect the samples. ZW, MK, and HL provided useful suggestions on the manuscript. All authors contributed to the manuscript revision, read and approved the submitted version.

## Conflict of Interest

The authors declare that the research was conducted in the absence of any commercial or financial relationships that could be construed as a potential conflict of interest.

## Supplementary Material

Supplementary Figure 1 The allelic richness (*R*), expected heterozygosity (*H*_E_), and number of private alleles (*P*_A_) over all nuclear SSR loci in each population of the seven dominant mangrove species along the coastline of South China.

Supplementary Figure 2 The correlations between Nei’s unbiased genetic distance (*G*_ST_) estimated by cpSSR loci and geographical distance of the seven dominant mangrove species along the coastline of South China.

Supplementary Figure 3 Correlation between genetic *F*_ST_/(1 − *F*_ST_) estimated by nuclear SSR loci and geographic distances among populations of the seven dominant mangrove species along the coastline of South China.

Supplementary Figure 4 The Delta *K* values (Delta *K* = mean[| L′′(*K*)|]/sd [L(*K*]) detected from STRUCTURE HARVESTER of seven dominant mangrove species: **(A)** *Acanthus ilicifolius*, **(B)** *Aegiceras corniculatum*, **(C)** *Avicennia marina*, **(D)** *Bruguiera gymnorrhiza*, **(E)** *Kandelia obovata*, **(F)** *Lumnitzera racemosa*, and **(G)** *Rhizophora stylosa*.

Supplementary Table 1 Chloroplast and nuclear microsatellite loci of the seven mangrove species used in this experiment.

Supplementary Table 2 Matrix of pairwise comparisons of population genetic differentiation (*F*_ST_) of the seven dominant mangrove species along the coastline of South China from nuclear SSR analysis.

Supplementary Table 3 Estimation of inter-population differentiation (*G*_ST_) and the number of substitution types (*N*_ST_) (mean ± SE in parentheses) of seven dominant mangrove species along the coastline of South China.

Supplementary Table 4 Recent migration rate between populations estimated from BAYESASS of seven dominant mangrove species along the coastline of South China from nuclear SSR analysis.

Supplementary Table 5 Mutation-scaled effective population size (Theta = 4Neμ) and mutation-scaled migration rate (M) estimated from Migrate-N of seven dominant mangrove species along the coastline of South China from nuclear SSR analysis.
