# Supplementary material for: Ocean Currents Drove Genetic Structure of Seven Dominant Mangrove Species Along the Coastlines of Southern China
Source: Front Genet. 2021 Mar 8;12:615911. doi: 10.3389/fgene.2021.615911 (PMC7982666; doi:10.3389/fgene.2021.615911)
Supplement: Supplementary Figure 1 — The allelic richness (R), expected heterozygosity (HE), and number of private alleles (PA) over all nuclear SSR loci in each population of the seven dominant mangrove species along the coastline of South China. [file Data_Sheet_1.docx]

**SUPPLEMENTARY INFORMATION**

**Figure S1｜**The allelic richness (*R*), expected heterozygosity (*H*_E_), and number of private alleles (*P*_A_) over all nuclear SSR loci in each population of the seven dominant mangrove species along the coastline of South China.

**Figure S2｜**The correlations between Nei's unbiased genetic distance (*G*_ST_) estimated by cpSSR loci and geographical distance of the seven dominant mangrove species along the coastline of South China.

**Figure S3｜**Correlation between genetic *F*_ST_/ (1- *F*_ST_) estimated by nuclear SSR loci and geographic distances among populations of the seven dominant mangrove species along the coastline of South China.

**Figure S4｜**The Delta *K* values (Delta *K*= mean[|L"(*K*)|]/ sd [L(*K*]) detected from STRUCTURE HARVESTER of seven dominant mangrove species: (a) *Acanthus ilicifolius*, (b) *Aegiceras corniculatum*, (c) *Avicennia marina*, (d) *Bruguiera gymnorrhiza*, (e) *Kandelia obovata*, (f) *Lumnitzera racemosa*, and (g) *Rhizophora stylosa*.

**Table S1｜**Chloroplast and nuclear microsatellite loci of the seven mangrove species used in this experiment.

**Table S2｜**Matrix of pairwise comparisons of population genetic differentiation (*F*_ST_) of the seven dominant mangrove species along the coastline of South China from nuclear SSR analysis.

**Table S3｜**Estimation of inter-population differentiation (*G*_ST_) and the number of substitution types (*N*_ST_)(mean ± SE in parentheses) of seven dominant mangrove species along the coastline of South China.

**Table S4｜**Recent migration rate between populations estimated from BAYESASS of seven dominant mangrove species along the coastline of South China from nuclear SSR analysis.

**Table S5｜**Mutation-scaled effective population size (Theta = 4Neμ) and mutation-scaled migration rate (M) estimated from Migrate-N of seven dominant mangrove species along the coastline of South China from nuclear SSR analysis.

**Figure S1｜**The allelic richness (*R*), expected heterozygosity (*H*_E_), and number of private alleles (*P*_A_) over all nuclear SSR loci in each population of the seven dominant mangrove species along the coastlines of South China.

**Figure S2｜**The correlations between Nei's unbiased genetic distance (*G*_ST_) estimated by cpSSR loci and geographical distance of the seven dominant mangrove species along the coastlines of South China.

**Figure S3｜**Correlation between genetic *F*_ST_/ (1- *F*_ST_) estimated by nuclear SSR loci and geographic distances among populations of the seven dominant mangrove species along the coastline of South China.

**Figure S4｜**The Delta *K* values (Delta *K*= mean[|L"(*K*)|]/ sd [L(*K*]) detected from STRUCTURE HARVESTER of seven dominant mangrove species: (a) *Acanthus ilicifolius*, (b) *Aegiceras corniculatum*, (c) *Avicennia marina*, (d) *Bruguiera gymnorrhiza*, (e) *Kandelia obovata*, (f) *Lumnitzera racemosa*, and (g) *Rhizophora stylosa*.

**Table S1｜**Chloroplast and nuclear microsatellite loci of the seven mangrove species used in this experiment.

| Species | Locus (reference) | |
| --- | --- | --- |
|  | Chloroplast | Nuclear |
| *Acanthus ilicifolius* | Acaicp01, Acaicp02, Acaicp03, Acaicp04, Acaicp05, Acaicp06, Acaicp07 (Geng *et al*. 2008) | Acil13, Acai02, Acai08, Acai11, Acai12, Acai13 (Geng *et al*. 2008) |
| *Aegiceras corniculatum* | Acorcp01, Acorcp02, Acorcp03, Acorcp04, Acorcp05 (Geng *et al*. 2008) | Acor02, Acor05, Acor07, Acor10, Acor12, Acor14, Acor15, Acor20, Acor26, Acor28 (Geng *et al*. 2006) |
| *Avicennia marina* | Avmacp01, Avmacp02, Avmacp03, Avmacp04, Avmacp05, Avmacp06, Avmacp07, Avmacp08, Avmacp09, Avmacp10, Avmacp11, Avmacp12, ccmp5 (Geng *et al*. 2008) | M3, M40, M47 (Maguire *et al*. 2000); Avma01, Avma02, Avma03, Avma05, Avma09, Avma14, Avma16, Avma17 (Geng *et al*. 2007) |
| *Bruguiera gymnorrhiza* | Brgycp01, Brgycp02, Brgycp03, Brgycp04, Brgycp05, Brgycp06, Brgycp07, Brgycp09, Brgycp10, Brgycp11, Rhstcp03 (Islam *et al*. 2008) | Brgy04, Brgy05, Brgy13, Brgy17, Brgy18, Brgy23, Brgy24, Brgy26 (Islam *et al*. 2006a) |
| *Kandelia obovata* | Kacacp07, Kacacp10, Kacacp11, Kacacp12, Kacacp14, Kacacp15, Kacacp16, Kacacp17 (Islam *et al*. 2008) | Kcan004, Kcan009, Kcan011, Kcan034, (Sugaya *et al*. 2002); Kaca01, Kaca04, Kaca05, Kaca09, Kaca10, Kaca12 (Islam *et al*. 2006b) |
| *Lumnitzera racemosa* | Lumrcp01, Lumrcp02, Lumrcp03, Lumrcp04, Lumrcp05, Lumrcp06, Lumrcp07, Lumrcp08, Lumrcp09, Lumrcp10, Lumrcp11, Lumrcp12, Lumrcp13, Lumrcp14, Lumrcp15, ccmp5 (Geng *et al*. 2008) | Lumr09, Lumr11, Lumr14, Lumr17, Lumr19, Lumr26, Lumr28, Lumr29, Lumr30 (Geng *et al*. 2008) |
| *Rhizophora stylosa* | Rhstcp01, Rhstcp02, Rhstcp03, Rhstcp04, Rhstcp05, Rhstcp06, Rhstcp07, Rhstcp08 (Islam *et al*. 2008) | Rhst01, Rhst02, Rhst11, Rhst13, Rhst15 (Islam *et al*. 2004); Rhst12, Rhst16, Rhst19, Rhst20, Rhst27 (Islam 2007) |

**Table S2｜**Matrix of pairwise comparisons of population genetic differentiation (*F*_ST_) of the seven dominant mangrove species along the coastline of South China from nuclear SSR analysis.

| Population no. | 1 | 2 | 3 | 4 | 5 | 6 | 7 | 8 | 9 | 10 | 11 | 12 | 13 | 14 | 15 | 16 | 17 | 18 | 19 | 20 |
| --- | --- | --- | --- | --- | --- | --- | --- | --- | --- | --- | --- | --- | --- | --- | --- | --- | --- | --- | --- | --- |
| *Acanthus ilicifolius* | | | | | | | | | | | | | | | | | | | | |
| 1 | 0.000 |  |  |  |  |  |  |  |  |  |  |  |  |  |  |  |  |  |  |  |
| 3 | 0.251 |  | 0.000 |  |  |  |  |  |  |  |  |  |  |  |  |  |  |  |  |  |
| 6 | 0.598 |  | 0.358 |  |  | 0.000 |  |  |  |  |  |  |  |  |  |  |  |  |  |  |
| 11 | 0.376 |  | 0.340 |  |  | 0.526 |  |  |  |  | 0.000 |  |  |  |  |  |  |  |  |  |
| 14 | 0.625 |  | 0.542 |  |  | 0.822 |  |  |  |  | 0.301 |  |  | 0.000 |  |  |  |  |  |  |
| 17 | 0.399 |  | 0.376 |  |  | 0.500 |  |  |  |  | 0.303 |  |  | 0.538 |  |  | 0.000 |  |  |  |
| *Aegiceras corniculatum* | | | | | | | | | | | | | | | | | | | | |
| 1 | 0.000 |  |  |  |  |  |  |  |  |  |  |  |  |  |  |  |  |  |  |  |
| 2 | 0.167 | 0.000 |  |  |  |  |  |  |  |  |  |  |  |  |  |  |  |  |  |  |
| 3 | 0.177 | 0.194 | 0.000 |  |  |  |  |  |  |  |  |  |  |  |  |  |  |  |  |  |
| 4 | 0.156 | 0.239 | 0.159 | 0.000 |  |  |  |  |  |  |  |  |  |  |  |  |  |  |  |  |
| 7 | 0.269 | 0.250 | 0.198 | 0.109 |  |  | 0.000 |  |  |  |  |  |  |  |  |  |  |  |  |  |
| 8 | 0.257 | 0.255 | 0.162 | 0.097 |  |  | 0.025 | 0.000 |  |  |  |  |  |  |  |  |  |  |  |  |
| 10 | 0.257 | 0.245 | 0.124 | 0.129 |  |  | 0.115 | 0.067 |  | 0.000 |  |  |  |  |  |  |  |  |  |  |
| 11 | 0.170 | 0.256 | 0.173 | 0.042 |  |  | 0.149 | 0.122 |  | 0.146 | 0.000 |  |  |  |  |  |  |  |  |  |
| 12 | 0.228 | 0.279 | 0.158 | 0.061 |  |  | 0.067 | 0.055 |  | 0.109 | 0.117 | 0.000 |  |  |  |  |  |  |  |  |
| 13 | 0.170 | 0.292 | 0.210 | 0.060 |  |  | 0.124 | 0.113 |  | 0.195 | 0.085 | 0.074 | 0.000 |  |  |  |  |  |  |  |
| 14 | 0.263 | 0.373 | 0.239 | 0.086 |  |  | 0.127 | 0.118 |  | 0.195 | 0.168 | 0.047 | 0.075 | 0.000 |  |  |  |  |  |  |
| 15 | 0.240 | 0.282 | 0.168 | 0.083 |  |  | 0.095 | 0.071 |  | 0.109 | 0.099 | 0.079 | 0.123 | 0.142 | 0.000 |  |  |  |  |  |
| 16 | 0.324 | 0.347 | 0.256 | 0.146 |  |  | 0.102 | 0.080 |  | 0.164 | 0.164 | 0.154 | 0.166 | 0.223 | 0.089 | 0.000 |  |  |  |  |
| 17 | 0.181 | 0.255 | 0.197 | 0.084 |  |  | 0.187 | 0.176 |  | 0.166 | 0.115 | 0.151 | 0.159 | 0.196 | 0.149 | 0.204 | 0.000 |  |  |  |

**Table S2｜**Continued

| Population no. | 1 | 2 | 3 | 4 | 5 | 6 | 7 | 8 | 9 | 10 | 11 | 12 | 13 | 14 | 15 | 16 | 17 | 18 | 19 | 20 |
| --- | --- | --- | --- | --- | --- | --- | --- | --- | --- | --- | --- | --- | --- | --- | --- | --- | --- | --- | --- | --- |
| *Avicennia marina* | | | | | | | | | | | | | | | | | | | | |
| 4 |  |  |  | 0.000 |  |  |  |  |  |  |  |  |  |  |  |  |  |  |  |  |
| 5 |  |  |  | 0.184 | 0.000 |  |  |  |  |  |  |  |  |  |  |  |  |  |  |  |
| 8 |  |  |  | 0.135 | 0.056 |  |  | 0.000 |  |  |  |  |  |  |  |  |  |  |  |  |
| 10 |  |  |  | 0.101 | 0.134 |  |  | 0.055 |  | 0.000 |  |  |  |  |  |  |  |  |  |  |
| 12 |  |  |  | 0.124 | 0.075 |  |  | 0.040 |  | 0.084 |  | 0.000 |  |  |  |  |  |  |  |  |
| 13 |  |  |  | 0.154 | 0.239 |  |  | 0.169 |  | 0.130 |  | 0.126 | 0.000 |  |  |  |  |  |  |  |
| 14 |  |  |  | 0.089 | 0.168 |  |  | 0.075 |  | 0.061 |  | 0.090 | 0.130 | 0.000 |  |  |  |  |  |  |
| 15 |  |  |  | 0.094 | 0.136 |  |  | 0.054 |  | 0.054 |  | 0.068 | 0.124 | 0.066 | 0.000 |  |  |  |  |  |
| 18 |  |  |  | 0.178 | 0.266 |  |  | 0.213 |  | 0.153 |  | 0.231 | 0.314 | 0.215 | 0.209 |  |  | 0.000 |  |  |
| 20 |  |  |  | 0.176 | 0.243 |  |  | 0.200 |  | 0.173 |  | 0.204 | 0.218 | 0.198 | 0.159 |  |  | 0.284 |  | 0.000 |
| *Bruguiera gymnorrhiza* | | | | | | | | | | | | | | | | | | | | |
| 3 |  |  | 0.000 |  |  |  |  |  |  |  |  |  |  |  |  |  |  |  |  |  |
| 7 |  |  | 0.030 |  |  |  | 0.000 |  |  |  |  |  |  |  |  |  |  |  |  |  |
| 8 |  |  | 0.071 |  |  |  | 0.044 | 0.000 |  |  |  |  |  |  |  |  |  |  |  |  |
| 9 |  |  | 0.036 |  |  |  | 0.032 | 0.018 | 0.000 |  |  |  |  |  |  |  |  |  |  |  |
| 10 |  |  | 0.047 |  |  |  | 0.045 | 0.022 | **0.004** | 0.000 |  |  |  |  |  |  |  |  |  |  |
| 11 |  |  | 0.144 |  |  |  | 0.162 | 0.240 | 0.199 | 0.225 | 0.000 |  |  |  |  |  |  |  |  |  |
| 14 |  |  | 0.121 |  |  |  | 0.058 | 0.097 | 0.103 | 0.125 | 0.211 |  |  | 0.000 |  |  |  |  |  |  |
| 17 |  |  | 0.071 |  |  |  | 0.069 | 0.094 | 0.085 | 0.098 | 0.110 |  |  | 0.090 |  |  | 0.000 |  |  |  |
| 18 |  |  | 0.168 |  |  |  | 0.152 | 0.189 | 0.163 | 0.169 | 0.254 |  |  | 0.182 |  |  | 0.106 | 0.000 |  |  |

**Table S2｜**Continued

| Population no. | 1 | 2 | 3 | 4 | 5 | 6 | 7 | 8 | 9 | 10 | 11 | 12 | 13 | 14 | 15 | 16 | 17 | 18 | 19 | 20 |
| --- | --- | --- | --- | --- | --- | --- | --- | --- | --- | --- | --- | --- | --- | --- | --- | --- | --- | --- | --- | --- |
| *Kandelia obovata* | | | | | | | | | | | | | | | | | | | | |
| 1 | 0.000 |  |  |  |  |  |  |  |  |  |  |  |  |  |  |  |  |  |  |  |
| 2 | 0.079 | 0.000 |  |  |  |  |  |  |  |  |  |  |  |  |  |  |  |  |  |  |
| 3 | 0.093 | 0.047 | 0.000 |  |  |  |  |  |  |  |  |  |  |  |  |  |  |  |  |  |
| 4 | 0.154 | 0.113 | 0.093 | 0.000 |  |  |  |  |  |  |  |  |  |  |  |  |  |  |  |  |
| 7 | 0.105 | 0.046 | 0.052 | 0.111 |  |  | 0.000 |  |  |  |  |  |  |  |  |  |  |  |  |  |
| 8 | 0.109 | 0.047 | 0.063 | 0.110 |  |  | 0.015 | 0.000 |  |  |  |  |  |  |  |  |  |  |  |  |
| 10 | 0.115 | 0.061 | 0.070 | 0.121 |  |  | 0.021 | 0.026 |  | 0.000 |  |  |  |  |  |  |  |  |  |  |
| 11 | 0.182 | 0.132 | 0.112 | 0.068 |  |  | 0.149 | 0.146 |  | 0.166 | 0.000 |  |  |  |  |  |  |  |  |  |
| 14 | 0.182 | 0.132 | 0.106 | 0.051 |  |  | 0.140 | 0.136 |  | 0.155 | 0.023 |  |  | 0.000 |  |  |  |  |  |  |
| 17 | 0.292 | 0.253 | 0.249 | 0.273 |  |  | 0.212 | 0.219 |  | 0.237 | 0.351 |  |  | 0.328 |  |  | 0.000 |  |  |  |
| *Lumnitzera racemosa* | | | | | | | | | | | | | | | | | | | | |
| 11 |  |  |  |  |  |  |  |  |  |  | 0.000 |  |  |  |  |  |  |  |  |  |
| 12 |  |  |  |  |  |  |  |  |  |  | 0.165 | 0.000 |  |  |  |  |  |  |  |  |
| 15 |  |  |  |  |  |  |  |  |  |  | 0.579 | 0.553 |  |  | 0.000 |  |  |  |  |  |
| 17 |  |  |  |  |  |  |  |  |  |  | 0.331 | 0.342 |  |  | 0.353 |  | 0.000 |  |  |  |
| 18 |  |  |  |  |  |  |  |  |  |  | 0.426 | 0.418 |  |  | 0.439 |  | 0.222 | 0.000 |  |  |
| 19 |  |  |  |  |  |  |  |  |  |  | 0.415 | 0.413 |  |  | 0.344 |  | 0.249 | 0.175 | 0.000 |  |

**Table S2｜**Continued

| Population no. | 1 | 2 | 3 | 4 | 5 | 6 | 7 | 8 | 9 | 10 | 11 | 12 | 13 | 14 | 15 | 16 | 17 | 18 | 19 | 20 |
| --- | --- | --- | --- | --- | --- | --- | --- | --- | --- | --- | --- | --- | --- | --- | --- | --- | --- | --- | --- | --- |
| *Rhizophora stylosa* | | | | | | | | | | | | | | | | | | | | |
| 3 |  |  | 0.000 |  |  |  |  |  |  |  |  |  |  |  |  |  |  |  |  |  |
| 4 |  |  | 0.259 | 0.000 |  |  |  |  |  |  |  |  |  |  |  |  |  |  |  |  |
| 5 |  |  | 0.144 | 0.120 | 0.000 |  |  |  |  |  |  |  |  |  |  |  |  |  |  |  |
| 7 |  |  | 0.254 | 0.282 | 0.208 |  | 0.000 |  |  |  |  |  |  |  |  |  |  |  |  |  |
| 8 |  |  | 0.141 | 0.122 | **0.007** |  | 0.216 | 0.000 |  |  |  |  |  |  |  |  |  |  |  |  |
| 9 |  |  | 0.071 | 0.131 | **0.020** |  | 0.188 | **0.015** | 0.000 |  |  |  |  |  |  |  |  |  |  |  |
| 11 |  |  | 0.425 | 0.065 | 0.189 |  | 0.392 | 0.190 | 0.226 |  | 0.000 |  |  |  |  |  |  |  |  |  |
| 12 |  |  | 0.554 | 0.213 | 0.280 |  | 0.558 | 0.304 | 0.291 |  | 0.340 | 0.000 |  |  |  |  |  |  |  |  |
| 13 |  |  | 0.131 | 0.067 | 0.049 |  | 0.156 | 0.056 | 0.047 |  | 0.144 | 0.197 | 0.000 |  |  |  |  |  |  |  |
| 14 |  |  | 0.117 | 0.089 | 0.024 |  | 0.195 | 0.022 | **0.020** |  | 0.182 | 0.251 | 0.040 | 0.000 |  |  |  |  |  |  |
| 15 |  |  | 0.133 | 0.125 | 0.050 |  | 0.215 | 0.057 | 0.035 |  | 0.237 | 0.190 | 0.031 | 0.040 | 0.000 |  |  |  |  |  |
| 16 |  |  | 0.345 | 0.278 | 0.286 |  | 0.330 | 0.308 | 0.259 |  | 0.412 | 0.414 | 0.183 | 0.248 | 0.186 | 0.000 |  |  |  |  |
| 17 |  |  | 0.281 | 0.147 | 0.088 |  | 0.323 | 0.111 | 0.119 |  | 0.223 | 0.281 | 0.136 | 0.105 | 0.138 | 0.349 | 0.000 |  |  |  |
| 18 |  |  | 0.683 | 0.540 | 0.624 |  | 0.671 | 0.633 | 0.613 |  | 0.624 | 0.671 | 0.561 | 0.598 | 0.599 | 0.600 | 0.609 | 0.000 |  |  |
| 20 |  |  | 0.747 | 0.585 | 0.680 |  | 0.733 | 0.691 | 0.668 |  | 0.680 | 0.733 | 0.609 | 0.652 | 0.652 | 0.652 | 0.666 | 0.071 |  | 0.000 |
| *F*_ST_ values in bold are non-significant; others are all significant (*P*< 0.05). | | | | | | | | | | | | | | | | | | | | |

**Table S3｜**Estimation of inter-population differentiation (Φ_ST_) and the number of substitution types (*N*_ST_, mean ± SE in parentheses) of seven dominant mangrove species in the coastlines of South China.

| Species | Φ_ST_ | *N*_ST_ |
| --- | --- | --- |
| *Acanthus ilicifolius* | 0.854 (0.0653) | 0.854 (0.0653) |
| *Aegiceras corniculatum* | 0.101 (nc) | 0.109 (nc) |
| *Avicennia marina* | 0.542 (0.1243) | 0.545 (0.1199) |
| *Bruguiera gymnorrhiza* | 0.219 (nc) | 0.243 (nc) |
| *Kandelia obovata* | 0.456 (0.1478) | 0.445 (0.1405) |
| *Lumnitzera racemosa* | 0.739 (0.1315) | 0.809 (0.1182) |
| *Rhizophora stylosa* | 0.805 (0.0311)* | 0.921 (0.0152)* |
| nc, not computed due to small sample size; *, *N*_ST_ is significantly different from *Φ*_ST_, *P* < 0.05 | | |

**Table S4｜** Recent migration rate between populations estimated from BAYESASS of seven dominant mangrove species in the coastlines of South China from nuclear SSR analysis.

| Source→Target↓ | 1 | 2 | 3 | 4 | 5 | 6 | 7 | 8 | 9 | 10 | 11 | 12 | 13 | 14 | 15 | 16 | 17 | 18 | 19 | 20 |
| --- | --- | --- | --- | --- | --- | --- | --- | --- | --- | --- | --- | --- | --- | --- | --- | --- | --- | --- | --- | --- |
| *Acanthus ilicifolius* | | | | | | | | | | | | | | | | | | | | |
| 1 |  |  | 0.0061 |  |  | 0.0061 |  |  |  |  | 0.0061 |  |  | 0.0061 |  |  | 0.0060 |  |  |  |
| 3 | 0.0130 |  |  |  |  | 0.0790 |  |  |  |  | 0.0128 |  |  | 0.0143 |  |  | 0.0129 |  |  |  |
| 6 | 0.0072 |  | 0.0073 |  |  |  |  |  |  |  | 0.0073 |  |  | 0.0073 |  |  | 0.0073 |  |  |  |
| 11 | 0.0073 |  | 0.0072 |  |  | 0.0073 |  |  |  |  |  |  |  | 0.0139 |  |  | 0.0073 |  |  |  |
| 14 | 0.0073 |  | 0.0072 |  |  | 0.0073 |  |  |  |  | 0.0076 |  |  |  |  |  | 0.0073 |  |  |  |
| 17 | 0.0073 |  | 0.0072 |  |  | 0.0073 |  |  |  |  | 0.0073 |  |  | 0.0073 |  |  |  |  |  |  |
| *Aegiceras corniculatum* | | | | | | | | | | | | | | | | | | | | |
| 1 |  | 0.0161 | 0.0062 | 0.0061 |  |  | 0.0061 | 0.0062 |  | 0.0062 | 0.0073 | 0.0062 | 0.0082 | 0.0064 | 0.0062 | 0.0061 | 0.0064 |  |  |  |
| 2 | 0.0077 |  | 0.0063 | 0.0061 |  |  | 0.0061 | 0.0061 |  | 0.0062 | 0.0063 | 0.0062 | 0.0064 | 0.0061 | 0.0064 | 0.0062 | 0.0063 |  |  |  |
| 3 | 0.0101 | 0.0105 |  | 0.01 |  |  | 0.0104 | 0.0107 |  | 0.0099 | 0.0143 | 0.0219 | 0.0102 | 0.0105 | 0.0103 | 0.0099 | 0.0115 |  |  |  |
| 4 | 0.0123 | 0.0061 | 0.0063 |  |  |  | 0.0064 | 0.0062 |  | 0.0062 | 0.2341 | 0.0108 | 0.0084 | 0.007 | 0.007 | 0.0064 | 0.0093 |  |  |  |
| 7 | 0.0063 | 0.0071 | 0.0064 | 0.0062 |  |  |  | 0.0066 |  | 0.0062 | 0.0066 | 0.009 | 0.0069 | 0.0223 | 0.0107 | 0.0131 | 0.0079 |  |  |  |
| 8 | 0.0061 | 0.0062 | 0.0062 | 0.0061 |  |  | 0.2284 |  |  | 0.0063 | 0.0071 | 0.0078 | 0.0094 | 0.0078 | 0.0132 | 0.0071 | 0.007 |  |  |  |
| 10 | 0.0145 | 0.0144 | 0.0147 | 0.0147 |  |  | 0.0779 | 0.0764 |  |  | 0.0148 | 0.0169 | 0.0144 | 0.0153 | 0.0153 | 0.0146 | 0.0148 |  |  |  |
| 11 | 0.0081 | 0.0066 | 0.0062 | 0.0062 |  |  | 0.007 | 0.0068 |  | 0.0062 |  | 0.007 | 0.1117 | 0.0073 | 0.0095 | 0.0078 | 0.018 |  |  |  |
| 12 | 0.0065 | 0.0064 | 0.0063 | 0.0062 |  |  | 0.0173 | 0.0073 |  | 0.0062 | 0.0176 |  | 0.0128 | 0.117 | 0.0094 | 0.0065 | 0.0071 |  |  |  |
| 13 | 0.0108 | 0.0064 | 0.0063 | 0.0062 |  |  | 0.009 | 0.0063 |  | 0.0063 | 0.0107 | 0.0083 |  | 0.1562 | 0.0076 | 0.0069 | 0.0069 |  |  |  |
| 14 | 0.0066 | 0.0062 | 0.0062 | 0.0062 |  |  | 0.0078 | 0.0065 |  | 0.0061 | 0.0084 | 0.0078 | 0.0082 |  | 0.0079 | 0.0064 | 0.0069 |  |  |  |
| 15 | 0.0104 | 0.0069 | 0.0062 | 0.0062 |  |  | 0.0138 | 0.0062 |  | 0.0061 | 0.0139 | 0.0085 | 0.0098 | 0.0313 |  | 0.0173 | 0.0096 |  |  |  |
| 16 | 0.0062 | 0.0063 | 0.0063 | 0.0061 |  |  | 0.018 | 0.0063 |  | 0.0062 | 0.0089 | 0.0079 | 0.0066 | 0.0113 | 0.0114 |  | 0.0068 |  |  |  |
| 17 | 0.007 | 0.0068 | 0.0079 | 0.0062 |  |  | 0.0071 | 0.0064 |  | 0.0063 | 0.0111 | 0.0098 | 0.0073 | 0.0115 | 0.0073 | 0.0066 |  |  |  |  |

**Table S4** Continued

| Source→Target↓ | 1 | 2 | 3 | 4 | 5 | 6 | 7 | 8 | 9 | 10 | 11 | 12 | 13 | 14 | 15 | 16 | 17 | 18 | 19 | 20 |
| --- | --- | --- | --- | --- | --- | --- | --- | --- | --- | --- | --- | --- | --- | --- | --- | --- | --- | --- | --- | --- |
| *Avicennia marina* | | | | | | | | | | | | | | | | | | | | |
| 4 |  |  |  |  | 0.0070 |  |  | 0.0078 |  | 0.0078 |  | 0.0067 | 0.0119 | 0.0110 | 0.0127 |  |  | 0.0068 |  | 0.0075 |
| 5 |  |  |  | 0.0069 |  |  |  | 0.0311 |  | 0.0084 |  | 0.0066 | 0.0067 | 0.0088 | 0.0081 |  |  | 0.0067 |  | 0.0069 |
| 8 |  |  |  | 0.0072 | 0.2130 |  |  |  |  | 0.0080 |  | 0.0068 | 0.0102 | 0.0085 | 0.0085 |  |  | 0.0068 |  | 0.0068 |
| 10 |  |  |  | 0.0086 | 0.0170 |  |  | 0.0237 |  |  |  | 0.0067 | 0.0327 | 0.0163 | 0.0218 |  |  | 0.0081 |  | 0.0072 |
| 12 |  |  |  | 0.0113 | 0.0206 |  |  | 0.1979 |  | 0.0112 |  |  | 0.0345 | 0.0130 | 0.0116 |  |  | 0.0110 |  | 0.0111 |
| 13 |  |  |  | 0.0081 | 0.0068 |  |  | 0.0080 |  | 0.0072 |  | 0.0066 |  | 0.0071 | 0.0095 |  |  | 0.0066 |  | 0.0067 |
| 14 |  |  |  | 0.0219 | 0.0150 |  |  | 0.0319 |  | 0.0084 |  | 0.0066 | 0.0175 |  | 0.0109 |  |  | 0.0068 |  | 0.0070 |
| 15 |  |  |  | 0.0099 | 0.0161 |  |  | 0.0944 |  | 0.0106 |  | 0.0068 | 0.0175 | 0.0200 |  |  |  | 0.0068 |  | 0.0070 |
| 18 |  |  |  | 0.0159 | 0.0078 |  |  | 0.0075 |  | 0.0081 |  | 0.0074 | 0.0074 | 0.0077 | 0.0075 |  |  |  |  | 0.0075 |
| 20 |  |  |  | 0.0085 | 0.0077 |  |  | 0.0078 |  | 0.0081 |  | 0.0075 | 0.0076 | 0.0083 | 0.0078 |  |  | 0.0088 |  |  |
| *Bruguiera gymnorrhiza* | | | | | | | | | | | | | | | | | | | | |
| 3 |  |  |  |  |  |  | 0.0561 | 0.0148 | 0.1433 | 0.0519 | 0.0141 |  |  | 0.0116 |  |  | 0.0171 | 0.0115 |  |  |
| 7 |  |  | 0.0073 |  |  |  |  | 0.0283 | 0.0687 | 0.1112 | 0.0107 |  |  | 0.0073 |  |  | 0.0125 | 0.0068 |  |  |
| 8 |  |  | 0.0070 |  |  |  | 0.0212 |  | 0.0492 | 0.1815 | 0.0089 |  |  | 0.0072 |  |  | 0.0141 | 0.0068 |  |  |
| 9 |  |  | 0.0071 |  |  |  | 0.0249 | 0.0425 |  | 0.1886 | 0.0107 |  |  | 0.0074 |  |  | 0.0102 | 0.0068 |  |  |
| 10 |  |  | 0.0073 |  |  |  | 0.0214 | 0.0326 | 0.0961 |  | 0.0095 |  |  | 0.0075 |  |  | 0.0125 | 0.0068 |  |  |
| 11 |  |  | 0.0072 |  |  |  | 0.0096 | 0.0079 | 0.0113 | 0.0092 |  |  |  | 0.0070 |  |  | 0.0201 | 0.0132 |  |  |
| 14 |  |  | 0.0202 |  |  |  | 0.1203 | 0.0247 | 0.0302 | 0.0293 | 0.0315 |  |  |  |  |  | 0.0332 | 0.0197 |  |  |
| 17 |  |  | 0.0082 |  |  |  | 0.0116 | 0.0116 | 0.0126 | 0.0136 | 0.0234 |  |  | 0.0083 |  |  |  | 0.0831 |  |  |
| 18 |  |  | 0.0215 |  |  |  | 0.0546 | 0.0227 | 0.0299 | 0.0268 | 0.0188 |  |  | 0.0212 |  |  | 0.0443 |  |  |  |

**Table S4** Continued

| Source→  Target↓ | 1 | 2 | 3 | 4 | 5 | 6 | 7 | 8 | 9 | 10 | 11 | 12 | 13 | 14 | 15 | 16 | 17 | 18 | 19 | 20 | |
| --- | --- | --- | --- | --- | --- | --- | --- | --- | --- | --- | --- | --- | --- | --- | --- | --- | --- | --- | --- | --- | --- |
| *Kandelia obovata* | | | | | | | | | | | | | | | | | | | | |  |
| 1 |  | 0.0133 | 0.0112 | 0.0143 |  |  | 0.0095 | 0.0096 |  | 0.0093 | 0.0094 |  |  | 0.0092 |  |  | 0.0092 |  |  |  | |
| 2 | 0.0088 |  | 0.0090 | 0.0079 |  |  | 0.0083 | 0.0081 |  | 0.0062 | 0.0081 |  |  | 0.0062 |  |  | 0.0062 |  |  |  | |
| 3 | 0.0353 | 0.0562 |  | 0.0182 |  |  | 0.0360 | 0.0177 |  | 0.0112 | 0.0333 |  |  | 0.0111 |  |  | 0.0112 |  |  |  | |
| 4 | 0.0072 | 0.0084 | 0.0180 |  |  |  | 0.0102 | 0.0108 |  | 0.0072 | 0.0745 |  |  | 0.0072 |  |  | 0.0070 |  |  |  | |
| 7 | 0.0065 | 0.0086 | 0.0103 | 0.0099 |  |  |  | 0.2126 |  | 0.0066 | 0.0080 |  |  | 0.0066 |  |  | 0.0069 |  |  |  | |
| 8 | 0.0072 | 0.0116 | 0.0076 | 0.0084 |  |  | 0.0138 |  |  | 0.0067 | 0.0120 |  |  | 0.0067 |  |  | 0.0067 |  |  |  | |
| 10 | 0.0088 | 0.0090 | 0.0088 | 0.0087 |  |  | 0.2484 | 0.0146 |  |  | 0.0087 |  |  | 0.0088 |  |  | 0.0088 |  |  |  | |
| 11 | 0.0066 | 0.0070 | 0.0066 | 0.0075 |  |  | 0.0069 | 0.0067 |  | 0.0066 |  |  |  | 0.0066 |  |  | 0.0066 |  |  |  | |
| 14 | 0.0081 | 0.0080 | 0.0081 | 0.0098 |  |  | 0.0081 | 0.0081 |  | 0.0082 | 0.2586 |  |  |  |  |  | 0.0081 |  |  |  | |
| 17 | 0.0105 | 0.0104 | 0.0103 | 0.0104 |  |  | 0.0103 | 0.0106 |  | 0.0105 | 0.0104 |  |  | 0.0103 |  |  |  |  |  |  | |
| *Lumnitzera racemosa* | | | | | | | | | | | | | | | | | | | | |  |
| 11 |  |  |  |  |  |  |  |  |  |  |  | 0.0490 |  |  | 0.0090 |  | 0.0090 | 0.0090 | 0.0090 |  | |
| 12 |  |  |  |  |  |  |  |  |  |  | 0.0096 |  |  |  | 0.0068 |  | 0.0068 | 0.0068 | 0.0069 |  | |
| 15 |  |  |  |  |  |  |  |  |  |  | 0.0119 | 0.0127 |  |  |  |  | 0.0118 | 0.0121 | 0.0120 |  | |
| 17 |  |  |  |  |  |  |  |  |  |  | 0.0101 | 0.0126 |  |  | 0.0087 |  |  | 0.0091 | 0.0079 |  | |
| 18 |  |  |  |  |  |  |  |  |  |  | 0.0072 | 0.0073 |  |  | 0.0079 |  | 0.0109 |  | 0.0208 |  | |
| 19 |  |  |  |  |  |  |  |  |  |  | 0.0073 | 0.0074 |  |  | 0.0085 |  | 0.0073 | 0.0219 |  |  | |

**Table S4** Continued

| Source→  Target↓ | 1 | 2 | 3 | 4 | 5 | 6 | 7 | 8 | 9 | 10 | 11 | 12 | 13 | 14 | 15 | 16 | 17 | 18 | 19 | 20 |  |
| --- | --- | --- | --- | --- | --- | --- | --- | --- | --- | --- | --- | --- | --- | --- | --- | --- | --- | --- | --- | --- | --- |
| *Rhizophora stylosa* | | | | | | | | | | | | | | | | | | | | | |
| 3 |  |  |  | 0.0096 | 0.1903 |  | 0.0096 | 0.0105 | 0.0096 |  | 0.0096 | 0.0096 | 0.0095 | 0.0126 | 0.0140 | 0.0097 | 0.0100 | 0.0096 |  | 0.0097 |  |
| 4 |  |  | 0.0084 |  | 0.0110 |  | 0.0083 | 0.0298 | 0.0084 |  | 0.0878 | 0.0257 | 0.0084 | 0.0321 | 0.0136 | 0.0085 | 0.0198 | 0.0082 |  | 0.0084 |  |
| 5 |  |  | 0.0068 | 0.0084 |  |  | 0.0068 | 0.0715 | 0.0067 |  | 0.0069 | 0.0115 | 0.0068 | 0.0098 | 0.0271 | 0.0072 | 0.0181 | 0.0067 |  | 0.0067 |  |
| 7 |  |  | 0.0134 | 0.0135 | 0.1346 |  |  | 0.0142 | 0.0134 |  | 0.0133 | 0.0133 | 0.0132 | 0.0201 | 0.0171 | 0.0134 | 0.0136 | 0.0133 |  | 0.0136 |  |
| 8 |  |  | 0.0061 | 0.0064 | 0.1776 |  | 0.0060 |  | 0.0061 |  | 0.0061 | 0.0100 | 0.0061 | 0.0093 | 0.0195 | 0.0063 | 0.0103 | 0.0060 |  | 0.0061 |  |
| 9 |  |  | 0.0061 | 0.0064 | 0.2031 |  | 0.0061 | 0.0206 |  |  | 0.0062 | 0.0067 | 0.0061 | 0.0091 | 0.0270 | 0.0064 | 0.0113 | 0.0062 |  | 0.0061 |  |
| 11 |  |  | 0.0061 | 0.0085 | 0.0072 |  | 0.0061 | 0.1311 | 0.0061 |  |  | 0.0064 | 0.0060 | 0.0070 | 0.0063 | 0.0060 | 0.0073 | 0.0061 |  | 0.0060 |  |
| 12 |  |  | 0.0061 | 0.0073 | 0.0073 |  | 0.0063 | 0.0095 | 0.0062 |  | 0.0063 |  | 0.0062 | 0.0114 | 0.0091 | 0.0063 | 0.0208 | 0.0062 |  | 0.0062 |  |
| 13 |  |  | 0.0060 | 0.0282 | 0.0606 |  | 0.0060 | 0.0109 | 0.0060 |  | 0.0359 | 0.0125 |  | 0.0239 | 0.1092 | 0.0066 | 0.0090 | 0.0060 |  | 0.0061 |  |
| 14 |  |  | 0.0060 | 0.0118 | 0.0953 |  | 0.0061 | 0.0355 | 0.0061 |  | 0.0102 | 0.0069 | 0.0064 |  | 0.0122 | 0.0064 | 0.0219 | 0.0062 |  | 0.0060 |  |
| 15 |  |  | 0.0060 | 0.0070 | 0.0400 |  | 0.0061 | 0.0717 | 0.0060 |  | 0.0061 | 0.0099 | 0.0061 | 0.0096 |  | 0.0188 | 0.0133 | 0.0060 |  | 0.0061 |  |
| 16 |  |  | 0.0061 | 0.0062 | 0.0073 |  | 0.0061 | 0.0062 | 0.0061 |  | 0.0061 | 0.0062 | 0.0061 | 0.0067 | 0.0428 |  | 0.0061 | 0.0061 |  | 0.0061 |  |
| 17 |  |  | 0.0061 | 0.0065 | 0.0400 |  | 0.0061 | 0.0270 | 0.0061 |  | 0.0061 | 0.0359 | 0.0060 | 0.0099 | 0.0107 | 0.0061 |  | 0.0061 |  | 0.0061 |  |
| 18 |  |  | 0.0060 | 0.0061 | 0.0061 |  | 0.0061 | 0.0060 | 0.0061 |  | 0.0060 | 0.0059 | 0.0061 | 0.0061 | 0.0060 | 0.0061 | 0.0061 |  |  | 0.1914 |  |
| 20 |  |  | 0.0061 | 0.0061 | 0.0062 |  | 0.0060 | 0.0060 | 0.0060 |  | 0.0061 | 0.0060 | 0.0061 | 0.0060 | 0.0061 | 0.0060 | 0.0061 | 0.0073 |  |  |  |
| For all values, the direction of migration is from the corresponding population in the first row to the corresponding population in the first column. | | | | | | | | | | | | | | | | | | | | | |

**Table S5** The mean number of migrants per generation (*N*_m_) estimated from Migrate-N of seven dominant mangrove species in the coastlines of South China.

| Source→  Target↓ | 1 | 2 | 3 | 4 | 5 | 6 | 7 | 8 | 9 | 10 | 11 | 12 | 13 | 14 | 15 | 16 | 17 | 18 | 19 | 20 |
| --- | --- | --- | --- | --- | --- | --- | --- | --- | --- | --- | --- | --- | --- | --- | --- | --- | --- | --- | --- | --- |
| *Acanthus ilicifolius* | | | | | | | | | | | | | | | | | | | | |
| 1 | - |  | 0.526 |  |  | 0.566 |  |  |  |  | 0.452 |  |  | 0.627 |  |  | 0.663 |  |  |  |
| 3 | 1.497 |  | - |  |  | 1.303 |  |  |  |  | 1.023 |  |  | 1.031 |  |  | 0.792 |  |  |  |
| 6 | 0.412 |  | 1.094 |  |  | - |  |  |  |  | 0.367 |  |  | 0.708 |  |  | 0.351 |  |  |  |
| 11 | 0.419 |  | 0.310 |  |  | 0.374 |  |  |  |  | - |  |  | 0.670 |  |  | 1.011 |  |  |  |
| 14 | 0.933 |  | 0.241 |  |  | 0.248 |  |  |  |  | 0.682 |  |  | - |  |  | 0.300 |  |  |  |
| 17 | 0.195 |  | 0.205 |  |  | 0.202 |  |  |  |  | 0.231 |  |  | 0.196 |  |  | - |  |  |  |
| *Aegiceras corniculatum* | | | | | | | | | | | | | | | | | | | | |
| 1 | - | 2.831 | 0.290 | 1.769 |  |  | 1.158 | 0.377 |  | 0.374 | 0.423 | 1.470 | 0.845 | 2.852 | 0.646 | 0.636 | 0.856 |  |  |  |
| 2 | 0.656 | - | 0.424 | 0.977 |  |  | 0.537 | 0.385 |  | 0.665 | 0.264 | 0.966 | 0.698 | 0.577 | 0.515 | 0.770 | 0.838 |  |  |  |
| 3 | 0.965 | 1.884 | - | 1.003 |  |  | 1.108 | 1.013 |  | 0.285 | 0.757 | 0.895 | 0.519 | 3.248 | 1.038 | 1.083 | 2.953 |  |  |  |
| 4 | 1.200 | 0.834 | 0.344 | - |  |  | 1.972 | 0.713 |  | 0.651 | 1.572 | 2.657 | 0.812 | 0.877 | 0.677 | 2.974 | 1.261 |  |  |  |
| 7 | 0.930 | 0.390 | 1.230 | 0.533 |  |  | - | 3.977 |  | 0.502 | 0.723 | 1.693 | 1.002 | 2.579 | 2.160 | 1.047 | 0.899 |  |  |  |
| 8 | 0.697 | 0.549 | 0.794 | 0.897 |  |  | 0.681 | - |  | 1.201 | 0.555 | 0.264 | 0.639 | 0.790 | 1.632 | 0.808 | 0.677 |  |  |  |
| 10 | 4.024 | 2.561 | 3.854 | 4.381 |  |  | 11.786 | 3.206 |  | - | 3.382 | 3.233 | 2.470 | 2.959 | 11.546 | 4.466 | 2.330 |  |  |  |
| 11 | 1.056 | 0.727 | 0.416 | 1.659 |  |  | 1.254 | 0.399 |  | 0.604 | - | 1.776 | 1.012 | 0.422 | 1.913 | 0.808 | 1.099 |  |  |  |
| 12 | 1.066 | 0.738 | 0.694 | 1.980 |  |  | 0.841 | 0.826 |  | 0.528 | 0.938 | - | 0.535 | 1.261 | 0.333 | 0.639 | 0.288 |  |  |  |
| 13 | 3.003 | 0.505 | 0.670 | 0.931 |  |  | 1.962 | 2.151 |  | 0.322 | 0.950 | 0.693 | - | 0.334 | 0.853 | 2.988 | 0.512 |  |  |  |
| 14 | 0.693 | 0.938 | 0.683 | 1.581 |  |  | 1.448 | 1.039 |  | 0.495 | 1.303 | 1.075 | 0.772 | - | 0.670 | 0.758 | 1.171 |  |  |  |
| 15 | 0.328 | 2.538 | 0.904 | 1.577 |  |  | 1.248 | 1.351 |  | 1.643 | 3.153 | 0.629 | 1.736 | 4.511 | - | 3.477 | 1.127 |  |  |  |
| 16 | 2.015 | 1.852 | 0.617 | 1.309 |  |  | 1.471 | 0.911 |  | 0.500 | 1.954 | 1.074 | 1.177 | 1.529 | 1.936 | - | 2.061 |  |  |  |
| 17 | 0.507 | 2.202 | 0.674 | 1.125 |  |  | 0.676 | 0.276 |  | 0.220 | 0.941 | 0.426 | 1.215 | 0.679 | 0.979 | 0.578 | - |  |  |  |

**Table S5** Continued

| Source→  Target↓ | 1 | 2 | 3 | 4 | 5 | 6 | 7 | 8 | 9 | 10 | 11 | 12 | 13 | 14 | 15 | 16 | 17 | 18 | 19 | 20 |
| --- | --- | --- | --- | --- | --- | --- | --- | --- | --- | --- | --- | --- | --- | --- | --- | --- | --- | --- | --- | --- |
| *Avicennia marina* | | | | | | | | | | | | | | | | | | | | |
| 4 |  |  |  | - | 1.397 |  |  | 1.087 |  | 1.861 |  | 0.816 | 1.081 | 1.145 | 1.647 |  |  | 1.905 |  | 0.990 |
| 5 |  |  |  | 0.926 | - |  |  | 0.935 |  | 1.005 |  | 1.033 | 1.102 | 1.760 | 1.242 |  |  | 1.039 |  | 1.079 |
| 8 |  |  |  | 1.866 | 2.867 |  |  | - |  | 1.289 |  | 2.865 | 1.921 | 2.733 | 1.452 |  |  | 1.410 |  | 0.524 |
| 10 |  |  |  | 2.449 | 2.351 |  |  | 2.363 |  | - |  | 1.978 | 2.484 | 2.923 | 2.749 |  |  | 0.677 |  | 2.240 |
| 12 |  |  |  | 3.700 | 1.520 |  |  | 6.567 |  | 5.792 |  | - | 2.300 | 3.403 | 3.872 |  |  | 2.138 |  | 1.224 |
| 13 |  |  |  | 1.428 | 0.848 |  |  | 1.204 |  | 3.238 |  | 0.942 | - | 0.984 | 0.526 |  |  | 0.813 |  | 0.314 |
| 14 |  |  |  | 2.376 | 2.299 |  |  | 2.614 |  | 2.229 |  | 2.205 | 6.558 | - | 1.670 |  |  | 2.512 |  | 2.643 |
| 15 |  |  |  | 2.804 | 2.151 |  |  | 2.888 |  | 2.137 |  | 1.574 | 1.757 | 3.786 | - |  |  | 1.842 |  | 4.793 |
| 18 |  |  |  | 2.948 | 0.982 |  |  | 1.808 |  | 0.959 |  | 0.765 | 1.199 | 1.195 | 1.740 |  |  | - |  | 1.505 |
| 20 |  |  |  | 1.370 | 1.818 |  |  | 0.720 |  | 1.012 |  | 1.504 | 1.762 | 4.414 | 2.553 |  |  | 2.326 |  | - |
| *Bruguiera gymnorrhiza* | | | | | | | | | | | | | | | | | | | | |
| 3 |  |  | - |  |  |  | 14.841 | 10.638 | 5.304 | 4.355 | 5.637 |  |  | 5.673 |  |  | 5.795 | 4.752 |  |  |
| 7 |  |  | 3.036 |  |  |  | - | 6.413 | 4.988 | 6.996 | 2.410 |  |  | 2.176 |  |  | 2.266 | 1.859 |  |  |
| 8 |  |  | 2.106 |  |  |  | 1.759 | - | 1.839 | 3.623 | 2.022 |  |  | 1.650 |  |  | 2.104 | 1.955 |  |  |
| 9 |  |  | 5.301 |  |  |  | 13.473 | 10.904 | - | 3.461 | 3.334 |  |  | 3.936 |  |  | 7.285 | 1.652 |  |  |
| 10 |  |  | 3.202 |  |  |  | 4.993 | 3.832 | 3.504 | - | 1.683 |  |  | 3.292 |  |  | 5.570 | 4.925 |  |  |
| 11 |  |  | 2.174 |  |  |  | 4.409 | 2.062 | 5.310 | 3.732 | - |  |  | 0.890 |  |  | 2.677 | 1.654 |  |  |
| 14 |  |  | 50.900 |  |  |  | 83.525 | 72.450 | 44.500 | 90.734 | 20.931 |  |  | - |  |  | 77.160 | 46.470 |  |  |
| 17 |  |  | 1.801 |  |  |  | 1.272 | 1.596 | 1.765 | 1.889 | 1.702 |  |  | 1.033 |  |  | - | 2.016 |  |  |
| 18 |  |  | 0.873 |  |  |  | 9.086 | 8.647 | 1.474 | 1.394 | 6.717 |  |  | 7.468 |  |  | 4.489 | - |  |  |

**Table S5** Continued

| Source→  Target↓ | 1 | 2 | 3 | 4 | 5 | 6 | 7 | 8 | 9 | 10 | 11 | 12 | 13 | 14 | 15 | 16 | 17 | 18 | 19 | 20 |
| --- | --- | --- | --- | --- | --- | --- | --- | --- | --- | --- | --- | --- | --- | --- | --- | --- | --- | --- | --- | --- |
| *Kandelia obovata* | | | | | | | | | | | | | | | | | | | | |
| 1 | - | 0.548 | 0.630 | 0.279 |  |  | 3.095 | 0.777 |  | 0.400 | 2.150 |  |  | 0.250 |  |  | 0.358 |  |  |  |
| 2 | 0.569 | - | 0.236 | 0.410 |  |  | 0.556 | 0.642 |  | 0.978 | 0.521 |  |  | 0.499 |  |  | 0.175 |  |  |  |
| 3 | 1.031 | 1.924 | - | 0.826 |  |  | 0.907 | 1.761 |  | 2.532 | 0.422 |  |  | 0.702 |  |  | 0.956 |  |  |  |
| 4 | 0.468 | 1.009 | 0.534 | - |  |  | 1.333 | 0.492 |  | 0.502 | 0.757 |  |  | 0.571 |  |  | 0.466 |  |  |  |
| 7 | 1.114 | 2.236 | 1.482 | 1.650 |  |  | - | 1.710 |  | 1.431 | 0.785 |  |  | 1.039 |  |  | 1.093 |  |  |  |
| 8 | 0.528 | 0.736 | 0.983 | 1.006 |  |  | 1.602 | - |  | 3.385 | 0.860 |  |  | 1.570 |  |  | 0.721 |  |  |  |
| 10 | 0.350 | 2.397 | 0.604 | 0.761 |  |  | 1.426 | 1.517 |  | - | 1.242 |  |  | 0.419 |  |  | 0.426 |  |  |  |
| 11 | 0.392 | 0.588 | 0.498 | 0.752 |  |  | 1.467 | 0.471 |  | 0.527 | - |  |  | 0.878 |  |  | 0.307 |  |  |  |
| 14 | 0.349 | 0.185 | 0.573 | 0.946 |  |  | 0.767 | 1.330 |  | 0.230 | 2.029 |  |  | - |  |  | 0.259 |  |  |  |
| 17 | 0.176 | 0.202 | 0.196 | 0.133 |  |  | 0.188 | 0.326 |  | 0.201 | 0.337 |  |  | 0.243 |  |  | - |  |  |  |
| *Lumnitzera racemosa* | | | | | | | | | | | | | | | | | | | | |
| 11 |  |  |  |  |  |  |  |  |  |  | - | 5.192 |  |  | 2.428 |  | 0.210 | 0.482 | 0.543 |  |
| 12 |  |  |  |  |  |  |  |  |  |  | 0.465 | - |  |  | 0.361 |  | 0.532 | 0.335 | 0.520 |  |
| 15 |  |  |  |  |  |  |  |  |  |  | 1.615 | 3.149 |  |  | - |  | 3.956 | 1.449 | 1.733 |  |
| 17 |  |  |  |  |  |  |  |  |  |  | 1.453 | 1.831 |  |  | 0.696 |  | - | 1.658 | 2.241 |  |
| 18 |  |  |  |  |  |  |  |  |  |  | 0.403 | 0.943 |  |  | 0.289 |  | 0.727 | - | 2.629 |  |
| 19 |  |  |  |  |  |  |  |  |  |  | 0.491 | 2.115 |  |  | 1.741 |  | 1.697 | 2.469 | - |  |

**Table S5** Continued

| Source→  Target↓ | 1 | 2 | 3 | 4 | 5 | 6 | 7 | 8 | 9 | 10 | 11 | 12 | 13 | 14 | 15 | 16 | 17 | 18 | 19 | 20 |
| --- | --- | --- | --- | --- | --- | --- | --- | --- | --- | --- | --- | --- | --- | --- | --- | --- | --- | --- | --- | --- |
| *Rhizophora stylosa* | | | | | | | | | | | | | | | | | | | | |
| 3 |  |  | - | 0.730 | 0.734 |  | 0.318 | 0.569 | 0.619 |  | 0.401 | 0.807 | 0.604 | 0.704 | 0.421 | 0.757 | 1.012 | 0.586 |  | 0.195 |
| 4 |  |  | 2.384 | - | 1.242 |  | 1.880 | 1.312 | 1.215 |  | 1.238 | 0.816 | 1.265 | 4.627 | 1.213 | 1.652 | 1.553 | 0.557 |  | 0.644 |
| 5 |  |  | 1.010 | 0.818 | - |  | 0.971 | 0.875 | 0.969 |  | 1.376 | 1.865 | 0.789 | 0.741 | 1.140 | 1.049 | 1.879 | 0.577 |  | 0.518 |
| 7 |  |  | 1.597 | 2.424 | 1.709 |  | - | 0.887 | 1.282 |  | 1.831 | 1.394 | 5.044 | 0.701 | 1.909 | 1.359 | 1.304 | 0.412 |  | 0.631 |
| 8 |  |  | 0.438 | 2.691 | 2.355 |  | 0.710 | - | 2.490 |  | 1.300 | 0.616 | 3.010 | 1.098 | 1.060 | 1.298 | 1.080 | 0.585 |  | 0.449 |
| 9 |  |  | 0.443 | 1.050 | 1.128 |  | 0.245 | 1.341 | - |  | 1.988 | 0.776 | 0.581 | 0.402 | 0.559 | 0.648 | 0.839 | 0.184 |  | 0.271 |
| 11 |  |  | 0.714 | 0.622 | 0.791 |  | 1.592 | 0.580 | 0.401 |  | - | 0.453 | 0.606 | 0.289 | 0.826 | 0.467 | 0.408 | 0.220 |  | 0.796 |
| 12 |  |  | 0.515 | 0.362 | 1.104 |  | 0.914 | 2.331 | 0.364 |  | 0.372 | - | 0.906 | 0.703 | 0.622 | 0.404 | 0.540 | 0.320 |  | 0.308 |
| 13 |  |  | 0.368 | 1.407 | 0.378 |  | 0.460 | 0.627 | 0.187 |  | 0.588 | 0.423 | - | 0.589 | 0.497 | 0.421 | 0.696 | 0.248 |  | 0.356 |
| 14 |  |  | 4.221 | 0.805 | 0.961 |  | 0.517 | 1.256 | 0.683 |  | 1.638 | 1.610 | 2.425 | - | 2.125 | 1.058 | 3.685 | 0.817 |  | 0.553 |
| 15 |  |  | 0.593 | 0.799 | 0.550 |  | 1.963 | 1.005 | 1.162 |  | 0.918 | 2.108 | 0.663 | 0.889 | - | 0.791 | 0.294 | 0.344 |  | 0.371 |
| 16 |  |  | 0.381 | 0.425 | 1.914 |  | 0.470 | 0.780 | 0.502 |  | 0.512 | 0.467 | 0.645 | 0.522 | 0.573 | - | 0.667 | 0.194 |  | 0.475 |
| 17 |  |  | 0.711 | 0.594 | 1.173 |  | 0.762 | 1.499 | 0.898 |  | 1.158 | 0.882 | 0.519 | 0.965 | 1.486 | 0.418 | - | 0.437 |  | 0.564 |
| 18 |  |  | 0.286 | 0.171 | 0.192 |  | 0.191 | 0.151 | 0.111 |  | 0.281 | 0.238 | 0.298 | 0.310 | 0.234 | 0.156 | 0.159 | - |  | 0.719 |
| 20 |  |  | 0.286 | 0.335 | 0.163 |  | 0.202 | 0.151 | 0.266 |  | 0.301 | 0.321 | 0.288 | 0.258 | 0.460 | 0.188 | 0.235 | 0.138 |  | - |

For all values, the direction of migration is from the corresponding population in the first row to the corresponding population in the first column.
